# Timing Constraints of *In Vivo* Gag Mutations during Primary HIV-1 Subtype C Infection

**DOI:** 10.1371/journal.pone.0007727

**Published:** 2009-11-05

**Authors:** Vladimir Novitsky, Rui Wang, Lauren Margolin, Jeannie Baca, Lemme Kebaabetswe, Raabya Rossenkhan, Caitlin Bonney, Michaela Herzig, David Nkwe, Sikhulile Moyo, Rosemary Musonda, Elias Woldegabriel, Erik van Widenfelt, Joseph Makhema, Stephen Lagakos, M. Essex

**Affiliations:** 1 Harvard School of Public Health AIDS Initiative, Department of Immunology and Infectious Diseases, Harvard School of Public Health, Boston, Massachusetts, United States of America; 2 Botswana–Harvard Partnership for HIV Research and Education, Gaborone, Botswana; 3 Department of Biostatistics, Harvard School of Public Health, Boston, Massachusetts, United States of America; Tsinghua University, China

## Abstract

**Background:**

Aiming to answer the broad question “When does mutation occur?” this study examined the time of appearance, dominance, and completeness of *in vivo* Gag mutations in primary HIV-1 subtype C infection.

**Methods:**

A primary HIV-1C infection cohort comprised of 8 acutely and 34 recently infected subjects were followed frequently up to 500 days post-seroconversion (p/s). Gag mutations were analyzed by employing single-genome amplification and direct sequencing. Gag mutations were determined in relation to the estimated time of seroconversion. Time of appearance, dominance, and completeness was compared for different types of *in vivo* Gag mutations.

**Results:**

Reverse mutations to the wild type appeared at a median (IQR) of 62 (44;139) days p/s, while escape mutations from the wild type appeared at 234 (169;326) days p/s (p<0.001). Within the subset of mutations that became dominant, reverse and escape mutations appeared at 54 (30;78) days p/s and 104 (47;198) days p/s, respectively (p<0.001). Among the mutations that reached completeness, reverse and escape mutations appeared at 54 (30;78) days p/s and 90 (44;196) days p/s, respectively (p = 0.006). Time of dominance for reverse mutations to and escape mutations from the wild type was 58 (44;105) days p/s and 219 (90;326) days p/s, respectively (p<0.001). Time of completeness for reverse and escape mutations was 152 (100;176) days p/s and 243 (101;370) days p/s, respectively (p = 0.001). Fitting a Cox proportional hazards model with frailties confirmed a significantly earlier time of appearance (hazard ratio (HR): 2.6; 95% CI: 2.3–3.0), dominance (4.8 (3.4–6.8)), and completeness (3.6 (2.3–5.5)) of reverse mutations to the wild type Gag than escape mutations from the wild type. Some complex mutational pathways in Gag included sequential series of reversions and escapes.

**Conclusions:**

The study identified the timing of different types of *in vivo* Gag mutations in primary HIV-1 subtype C infection in relation to the estimated time of seroconversion. Overall, the *in vivo* reverse mutations to the wild type occurred significantly earlier than escape mutations from the wild type. This shorter time to incidence of reverse mutations remained in the subsets of *in vivo* Gag mutations that reached dominance or completeness.

## Introduction

Knowledge of early events in infection is essential for understanding HIV-1 pathogenesis. Although correlates of protection in HIV-1 infection are still elusive, it is widely believed that the breadth of immune response plays an important role in disease outcome, and is likely to be associated with the breadth of viral mutational pathways. In natural HIV-1 infection, viral mutational pathways that are not directly related to ART are still poorly understood. This is because in most studies the time of infection is rarely available, which makes synchronizing viral mutations extremely difficult. In contrast, when the time of infection or seroconversion can be reliably identified [1]–[4], the time course of these mutations relative to infection or seroconversion can be established. To date, surprisingly little attention has been devoted to the timing of viral mutations in the natural course of HIV infection. It is likely that knowledge of timing constraints on viral mutations could shape preventive and therapeutic strategies by identifying optimal times of intervention.

Viral escape mutations in the natural course of HIV infection have been extensively studied [3]–[16], and were primarily associated with virus-specific CD8+ T cell responses. A recent report by Goonetilleke at al. [17] provided evidence that the T cell responses to transmitted virus can occur very early, and are accountable for escape mutations during the drop of viral load peak in the acute phase of HIV infection. Escape mutations accumulated over the course of HIV infection can revert to HIV-1 consensus upon transmission to a new host with a different genetic makeup. In the absence of immune pressure, reverse mutations to the HIV-1 subtype consensus are assumed to restore viral fitness that was apparently reduced due to escape mutations in the previous host. It is believed that reverse mutations to subtype consensus are widely observed during the early phase of HIV-1 infection [2], [13], [15], [18], [19], although quantitative and timing relationships between reverse and escape mutations are uncertain. In particular, little is known about the transient nature of HIV-1 mutations.

HIV-1 Gag is one of the most attractive targets for vaccine design, as it is able to induce potent virus-specific T cell responses associated with control of viral replication, lower viral set point, and more favorable disease prognosis [20]–[32]. The timing of viral mutational pathways and the impact of specific time of viral mutations on virus-specific T cell responses are still unclear. Therefore, a better understanding of viral mutational pathways in Gag and their temporal constraints may advance rational design of an HIV-1 vaccine.

To address the question of when *in vivo* Gag mutations occur, we focused on examining the time of appearance, dominance, completeness, and loss of different types of viral mutations in Gag soon after seroconversion over the first year of HIV-1 subtype C infection in a cohort of 42 subjects with estimated time of seroconversion. Such information may be useful in the design of comprehensive immunologic studies, and set the stage for future studies aimed at analysis of adaptive immune responses causing or responding to viral mutations.

## Methods

### Ethics Statement

This study was conducted according to the principles expressed in the Declaration of Helsinki. The study was approved by the Institutional Review Boards of Botswana and the Harvard School of Public Health. All patients provided written informed consent for the collection of samples and subsequent analysis.

### Study Subjects

**Figure 1:**
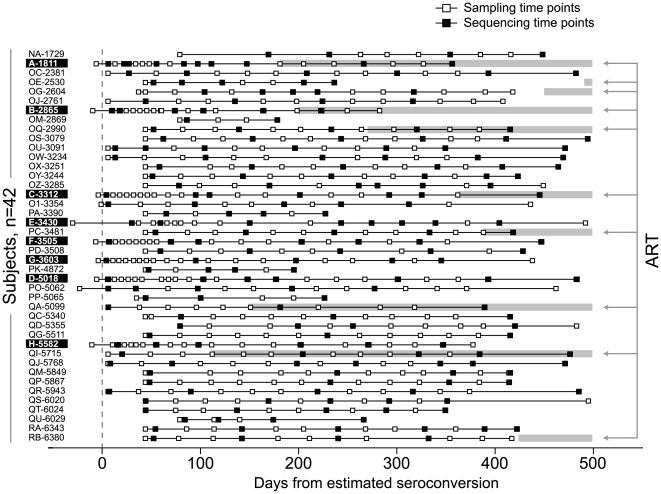
Time points of sampling (open squares) and sequencing (filled squares) in the study. A time scale is set to the estimated time of seroconversion as time 0. The sampling time points were limited to 500 days p/s. The study subjects' code is shown in the column at the left. Acutely infected subjects are highlighted. Ten subjects who initiated ART are delineated by gray bars and arrows on the right.

Subjects with acute and recent HIV infection were enrolled in a primary HIV-1 subtype C infection cohort in Botswana [33], [34] from April 2004 to April 2008. Forty-two subjects included 8 acutely infected (Fiebig stage II) and 34 recently infected (Fiebig stage IV or V) individuals (Figure 1). Time of seroconversion (time zero) for acutely infected subjects was estimated as the midpoint between the last seronegative test and the first seropositive test (within a week in most cases), and for recently infected subjects by Fiebig staging [35]. Details on assignment of Fiebig stage are described elsewhere [36] and provided in Figure S1. The cohort included 9 male (2 acute) and 33 female (6 acute) participants. The median age at enrollment was 27 years (IQR 25–32, range 20–56). All subjects were Botswana nationals, and all infections were HIV-1 subtype C [34], [37]. Viral load and CD4+ T cell counts were assessed during follow-up [33], [34], [37]. The median follow up period was 378 (IQR 321–447) days post-seroconversion (p/s). ART was initiated in 10 of 42 (24%) subjects within the observed period of time due to a drop in CD4+ T cells.

### Single-Genome Amplification and Sequencing

Previous studies showed comparability of the HIV-1 sequences originated from cell-associated proviral DNA and viral RNA from plasma [1], [38]. In this study both viral RNA from plasma and cell-associated proviral DNA were used as sources of templates for amplification of viral sequences. Viral RNA was isolated from plasma by QIAamp viral RNA Mini kit or QIAamp UltraSens Virus kit (Qiagen, Valencia, CA). The proviral DNA was isolated from buffy coats or PBMC by QIAamp DNA Mini kit (Qiagen, Valencia, CA). The cDNA synthesis was performed using the Transcriptor First Strand cDNA Synthesis kit (Roche Diagnostics, Indianapolis, IN) and minus-strand primer gagDrev[39] (5′- AAT TCC TCC TAT CAT TTT TGG; HXB2 nucleotide positions 2,382 to 2,402). Both cDNA and DNA templates were subjects for single-genome amplification by limiting dilutions [40], [41] with some modifications. The analyzed region of *gag* corresponded to nucleotide positions 841 to 2,217 of HXB2 (amino acids 18 to 476 in relation to the Gag CDS in HXB2). FastStart High Fidelity Enzyme Blend (Roche Diagnostics, Indianapolis, IN) was used in both rounds of PCR. Primers F2NST [42] (5′-GCG GAG GCT AGA AGG AGA GAG ATG G; HXB2 nucleotide positions 769 to 793) and 1448L (5′-AGG GGT CGC TGC CAA AGA GTG ATT; HXB2 nucleotide positions 2,258 to 2,291) were used in the 1^st^ round PCR. Primers gag-5U (5′-GTG CGA GAG CGT CAA TAT TAA GAG; HXB2 nucleotide positions 794 to 817) and 1445L (5′-GGT CGC TGC CAA AGA GTG ATT; HXB2 nucleotide positions 2,258 to 2,278) were used in the 2^nd^ round PCR. Amplicons from dilutions with fewer than 30% positive reactions were purified by Exo-SAP [43], and were sequenced directly at both strands on the ABI 3730 DNA Analyzer using BigDye technology. Sequence contigs were assembled by SeqScape v.2.6. Multiple sequence alignment was performed by HIV-align at the Los Alamos HIV Database site (http://www.hiv.lanl.gov/) using the hidden Markov model and the codon-alignment option followed by minor manual adjustments in BioEdit [44]. The obtained sequences were tested by HYPERMUT v.2.0 [45] and hypermutated sequences were excluded from analysis. The median analyzed viral quasispecies in Gag (IQR) was 58.5 (42.0; 72.8) per subject, and 11.1 (8.8; 13.6) viral sequences per time point per subject. Sequences from ARV-treated subjects were included in the analysis and their corresponding time points are shown by gray bars in Figure 1. The number of *gag* sequences per time point per patient indicating the number of RNA and DNA sequences generated in the study is presented in Figure S1. The relatively limited number of sequenced quasispecies does not allow us to exclude potential artifacts of sequencing, and it is possible that at least some transient or minor mutations observed in this study are not true mutations. To address the potential similarity between RNA and DNA in primary HIV infection the phylogenetic relationships between RNA and DNA sequences obtained from the same sampling time points were analyzed. We found no evidence for independent clustering, and the pattern of intermingling between analyzed RNA and DNA sequences was overwhelming (data not shown). Therefore, we included both RNA and DNA sequences in the analysis.

### Transmitted Viruses

To determine multiplicity of infection we used the methodology described by Keele et al. [46], and found that 12 out of 42 cases (29%; including one acutely infected subject) were likely to be infected with distinct viral variants as evident by the maximum achievable *gag* diversity of more than 1% and maximum achievable number of differences (Hamming distances) of 14 or more. A subset of 7 subjects (17%; including 2 acutely infected subjects) were likely to be infected with a few closely related variants. Thus, 30 out of 42 subjects (71%) in this study were infected with a single or closely related viral variants.

### Frequency of Viral Quasispecies

**Figure 2:**
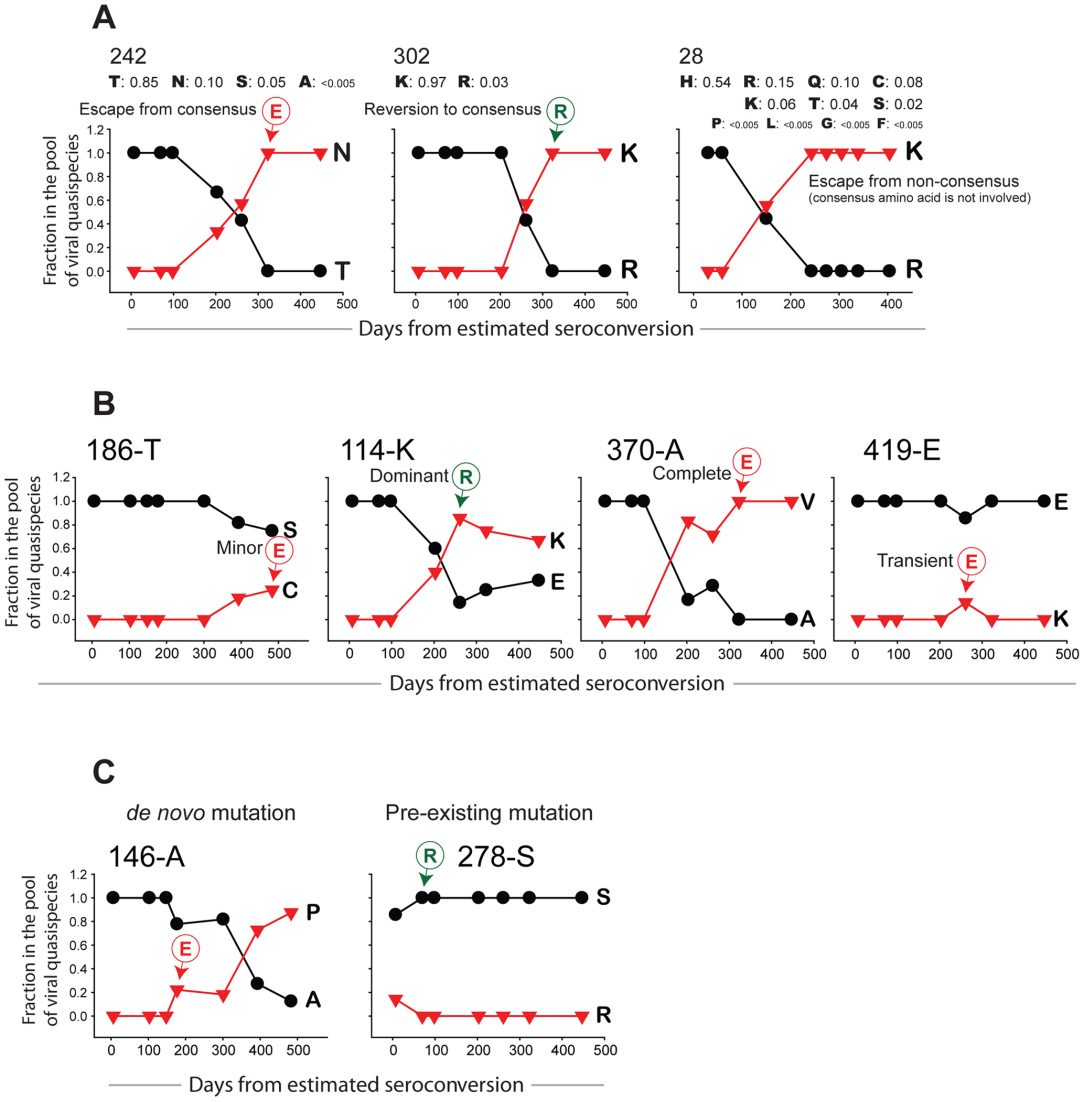
Types of analyzed amino acid substitutions in the pool of viral quasispecies over time. **A:** Viral mutations by their relationship to HIV-1 wild type. Number at the top of each graph indicates amino acid position in Gag, and is followed by frequencies of observed amino acids in the extended HIV-1 subtype C consensus. Dynamics of Asn at amino acid position 242 represents escape mutation from the wild type virus, and is delineated by circled E with arrow. Kinetics of Lys at position 302 demonstrates reverse mutation to the wild type virus. Viral mutation at position 28, Arg-to-Lys substitution, occurs without involvement of the wild type virus, and therefore, is interpreted as escape from non-wild type. **B:** Viral mutations by their fraction in the pool of viral quasispecies are classified as minor (position 186), dominant (position 114), complete (position 370), or transient (position 419). Number at the top of each graph stays for the amino acid position in Gag followed by the most common amino acids in the HIV-1 subtype C consensus, which is used to identify escape from and reversions to wild type virus. Circled letters with arrows indicate reverse mutations to and escape mutations from HIV-1 subtype C wild type. **C:** Viral mutations by their presence in the earliest pool of viral quasispecies are classified as *de novo* mutation (position 146), or pre-existing mutation (position 278). Circled letters with arrows indicate escape or reverse mutations.

This study was focused on non-synonymous substitutions in Gag only at time points presented in Figure 1 (and outlined in Figure S1). The frequencies of translated amino acid sequences were analyzed using MargFreq [47] per time point per subject. The frequency of each amino acid was expressed as a fraction of 1 in the pool of viral quasispecies at a given time point. Evolution of amino acid frequencies was plotted over time in relation to the estimated time of seroconversion as time 0. Dynamics of *in vivo* Gag mutations was the primary focus of this study. Any change in the proportion of amino acid(s) in the pool of viral quasispecies over time was treated as mutation. At each analyzed amino acid residue, we focused only on the amino acid with increasing proportion in the pool of viral quasispecies over time. Therefore, frequency changes in the pool of viral quasispecies over time were tracked according to the following criteria based on their presence in the earliest available sample. Sites with a single amino acid present in the pool of the earliest available quasispecies were examined for appearance of any alternative amino acid over the time of follow up, and the new amino acid was subject to evaluation. For example, according to the viral dynamics at Gag position 242 in Figure 2A, only Thr was present in the pool of viral quasispecies at the earliest time point at 7 days p/s (and also at day 70 p/s and day 98 p/s), while the first appearance of alternative Asn was observed at day 203 p/s. Therefore, the kinetics of Asn was a subject for evaluation at this site. In contrast, if multiple amino acids were present in the pool of the earliest available quasispecies, the amino acid that showed increase in its frequency over time was a subject for analysis. For example, due to the dynamics of viral mutations at Gag position 278 in Figure 2C, Ser was a subject for evaluation and analysis. The evolution of amino acid frequencies was plotted over time in relation to the estimated time of seroconversion as time 0.

### Types of Amino Acid Substitutions in Gag

The main focus of the study was on the time from estimated seroconversion necessary to reach a specific state of viral mutation. All observed mutations were included in the analysis. States of viral mutations in Gag were analyzed at each amino acid position over time, and classified by (i) their relation to wild type virus (subtype consensus), (ii) fraction in the pool of viral quasispecies, and (iii) presence at earliest time point. The most common viral variant circulating within the given host population, or wild type, is assumed to be the optimally fit virus which is commonly expressed as an HIV-1 subtype consensus sequence [13], [17], [48], [49]. The term wild type virus corresponds to HIV-1 subtype C consensus throughout the manuscript. The observed mutations from the wild type are classified as *potential* escape mutations, and appropriate validation by immunological assays might be necessary to prove their escaping nature. Two types of escape mutation were distinguished: escape from the wild type (Figure 2A, position 242), and escape from non-wild type (Fig. 2A, position 28). A mutation toward the wild type was considered a reverse mutation, or reversion (Figure 2A, position 302).Two meanings of the term *reverse mutation*—narrow and broader—should be noted. The narrow definition of the term *reverse mutation* denotes reversion to the ancestral state of transmitted virus, and applies to a limited number of hosts linked by virus transmission. Examples of the narrow use of the term include but are not limited to traceable chains of viral transmission (e.g., confirmed donor–recipient pairs in the case of HIV-discordant couples), or cases where transmitted virus can be reliably reconstructed due to early sampling (e.g., Fiebig stage I or II) and series of *in vitro* experiments. The second, broader definition of the term *reverse mutation*, which is widely used in molecular epidemiology studies addressing viral evolution on the population level, stands for reversion to the wild type virus in the absence of immune pressure upon transmission to a new host [2], [4], [48], [50], [51]. This definition relies on several assumptions postulated in a series of recent studies [2], [13], [15], [16], [18], [19], [49], [52], [53] including the following: *i*) the wild type virus corresponds to HIV-1 subtype consensus sequence; *ii*) the wild type virus is the most fit viral variant, at least in the local epidemic environment; *iii*) escape mutation(s) in the previous host occurred before transmission to a new host; *iv*) reverse mutation represents a restoration of viral fitness in the absence of immune pressure; and *v*) a mis-match of genetic background between previous and new hosts is expected, particularly in the profile of MHC class I HLA alleles. The second definition of the term *reverse mutation*, denoting mutation toward HIV subtype consensus, is used throughout the current study.

Based on the frequency of specific amino acids in the pool of viral quasispecies, we distinguished *minor* (Figure 2B, position 186), *dominant* (Figure 2B, position 114), *complete* (Figure 2B, position 370), and *transient* (Figure 2B, position 419) amino acid substitutions. A mutation was considered *minor* if its fraction was less than or equal to 50% in the pool of viral quasispecies during follow-up; e.g., a minor escape mutation is shown at position 186 in Figure 2B. A mutation was treated as *dominant* if its fraction reached more than 50% in the pool of viral quasispecies but did not reach complete substitution of original amino acid; e.g., a dominant reverse mutation is shown at position 114 in Figure 2B. A mutation was *complete* if it reached 100% in the pool of viral quasispecies during the follow-up period; e.g., a complete escape mutation is presented at position 370 in Figure 2B. A mutation was considered *transient* if its frequency subsequently decreased below 100% for complete mutations, or below 50% for dominant mutations, or if minor mutations disappeared over the observation period; e.g, a minor transient mutation is outlined at position 419 in Figure 2B.

Based on the presence or absence of a particular viral variant in the pool of viral quasispecies at the earliest available time point we distinguished *de novo* (Figure 2C, position 146) and *pre-existing* (Figure 2C, position 278) viral mutations. That a mutation is pre-existing does not necessarily mean that it was transmitted because early immune response may contribute to early viral diversification. Of note, true *de novo* and pre-existing status of viral mutations can be identified if sampling was performed at a very early time point, e.g., in Fiebig stage I or II before the earliest immune responses occur [17], [46]. Therefore, in this study identification of true *de novo* and pre-existing state of viral mutations can be reliably applied to eight cases of acute infection, but should be taken cautiously for the subset of 34 recently infected subjects, particularly for individuals sampled in Fiebig stage V.

### MHC Class I HLA Typing

High resolution HLA typing was performed on all 42 subjects in the study using the AlleleSEQR HLA Sequencing-Based Typing kit (Celera, Alameda, CA) according to the manufacturer's instruction. Contig assembling and assignment of HLA alleles was implemented by Assign SBT ver. 3.5.1.42 (Conexio Genomics, Applecross, Australia). All ambiguous positions were resolved by re-sequencing. Polymorphisms outside the targeted exons that could not resolve heterozygote combinations are presented as HLA allele variants in brackets or as two-digit HLA typing results.

### Statistical Analysis

Timing data are summarized with medians (interquartile range for 25% and 75%). If the number of viral mutations was sufficient (n≥3), comparisons of continuous outcomes between two groups were based on Mann-Whitney Rank Sum tests. Cox proportional hazards models with frailties were used to assess and compare the time until different types of amino acid substitutions, while taking into account correlations among timing data contributed by the same subject. All reported p-values are 2-sided and not adjusted for multiple analyses.

### Nucleotide Sequence Accession Numbers

A total of 2,522 *gag* sequences generated in this study were deposited in GenBank. The accession numbers are GQ275380–GQ277569, GQ375107–GQ375128, and GQ870874–GQ871183.

## Results

### Heterogeneous Evolution of Gag Quasispecies in HIV-1 Subtype C Infected Subjects

**Figure 3:**
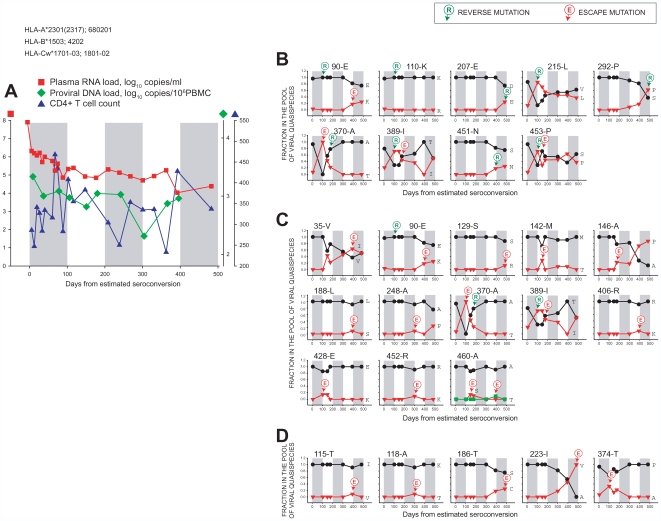
Temporal relationships between frequency distributions of Gag quasispecies in acutely infected subject D-5018 from day 6 p/s to day 483 p/s. MHC class I HLA profile is presented at the top of the figure. Gray bars at the background of each graph delineate 100-days time intervals for better comparison between dynamics of viral load/CD4 (Figure 3A), and time of Gag mutations (Figures 3B to 3D). Number with the letter at the top of each graph in B, C, and D represents amino acid position in Gag according to the HXB2 numbering (http://www.hiv.lanl.gov/) and the most frequent amino acid in the HIV-1 subtype C consensus. Green circled R with arrow delineates reverse mutation to wild type virus, and red circled E with arrow indicates escape mutations from wild type. **A:** Dynamics of plasma RNA load (red squares), proviral DNA load (green diamonds), and CD4+ T cell count (blue triangles) over the follow up period. **B:** Reverse mutations to HIV-1 subtype C wild type virus. **C:** Escape mutations from HIV-1 subtype C wild type virus. D: Escape mutations from non-wild type (wild type amino acid is not involved).

All analyses in this study were performed using the cohort of 42 subjects with estimated time of seroconversion. Figures 3 to [Fig pone-0007727-g004] 5 exemplify the evolution of *in vivo* Gag mutations per amino acid position in three subjects identified with acute HIV-1 subtype C infection. In addition, Figure S2, S3, S4, S5, S6 show evolution of *in vivo* Gag mutations in five other acutely infected subjects, and Figures S7 and S8 show alignments of translated amino acids for two acutely infected subjects, D-5018 and E-3430, respectively. Data from 34 recently infected subjected is not shown, although all the data were included in the analyses. The following three examples highlight a remarkable heterogeneity in the evolution of *in vivo* Gag mutations between subjects during the early phase of HIV-1 subtype C infection.

**Figure 4 pone-0007727-g004:**
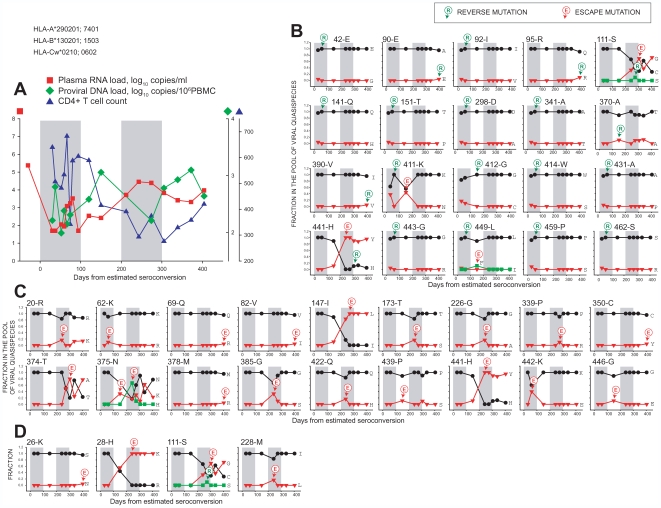
Temporal relationships between frequency distributions of Gag quasispecies in acutely infected subject E-3430 from day 30 p/s to day 404 p/s. See legend to Figure 3.

**Figure 5:**
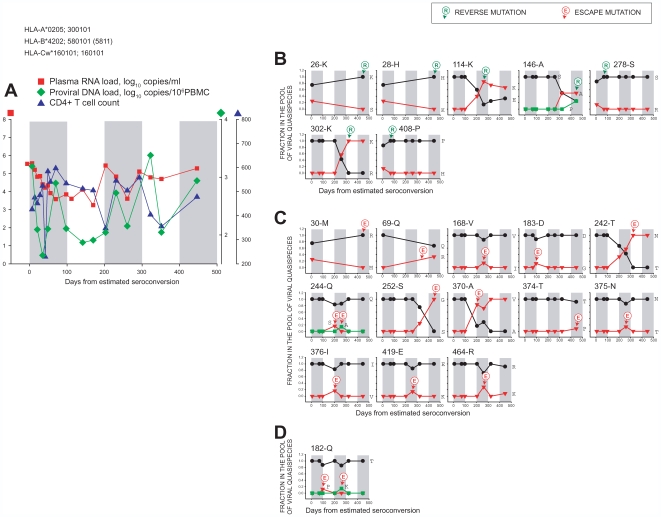
Temporal relationships between frequency distributions of Gag quasispecies in acutely infected subject F-3505 from day 7 to 447 days p/s. See legend to Figure 3. Amplicons from time points from day 70 p/s to day 323 p/s were amplified with alternative primers which resulted in excluding the first 109 amino acids (HXB2 numbering; http://www.hiv.lanl.gov/) from analysis, and is reflected in the graphs at amino acid positions 26 and 28 (Figure 5B), and 30 and 69 (Figure 5C).

Kinetics of viral RNA load, proviral DNA load, and CD4+ T cell count in subject D-5018 are presented in Figure 3A, while dynamics of Gag mutations are shown in Figures 3B (reverse mutations), 3C (escape mutations from consensus), and 3D (escape from non-wild type). A dramatic fluctuation of CD4+ T cell count around 100 days p/s (Figure 3A) coincided with dynamic changes in the spectrum of viral quasispecies associated with reverse mutations to the wild type (Figure 3B; amino acid positions 215, 389, and 453) and escape mutations from the wild type (Figure 3C; amino acid positions 35, 142, 146, 370, and 428). Viral RNA load in subject D-5018 remained high at the level of 5.0 log_10_ copies/ml for about a year p/s. Viral load was reduced about 1.0 log_10_ around 400 days p/s, and was accompanied by reverse mutations at Gag positions 207, 292, 451 (Figure 3B), escape mutations from the wild type at positions 90 and 129 (Figure 3C), and escape mutations from non-wild type at positions 186 and 223 (Figure 3D). However the relationship between observed viral mutations and viral load changes and their causality in subject D-5018 should be taken cautiously without support of functional immunologic data. At Gag position 90, the interplay of two amino acids was observed from day 6 p/s to day 483 p/s. At day 6 p/s, this amino acid position was shared between Glu at frequency 0.97 and Lys at 0.03, but then the frequency of Glu increased to 1.0 at days 103, 148, 177, and 301 p/s providing evidence for a complete reversion to wild type and eliminating Lys from the pool of viral quasispecies. However at days 393 and 483 p/s Lys was present at a frequency of 0.18 and 0.25, respectively. Dynamics of viral quasispecies at position 90 revealed a phenomenon of reverse mutation to wild type virus followed by escape mutation from the wild type. Interestingly, some of the dominant mutations lost their dominance at later time points, although as is evident from the example of subject D-5018, timing of loss was unique for each amino acid position (Figure 3). Leu at position 215 in p24 was a minor amino acid at frequency 0.14 by day 6 p/s, and its fraction increased to 0.86, suggesting a dominance at day 103 p/s. However the fraction of Leu was gradually reduced to 0.71, 0.56, 0.45, 0.45, and 0.38 at days 148, 177, 301, 393, and 483 p/s, respectively, suggesting a balance being established between Leu and Val at later time points. Ile at position 389 located in the p7 region of Gag stayed at frequency 0.21 by day 6 p/s, reached 0.71 at day 103 p/s, and maintained dominance at day 148 p/s, but then dropped to 0.44, 0.36, 0, and 0.50 at days 177, 301, 393, and 483 p/s, respectively, also suggesting a balance between Ile and Thr similar to the scenario seen at position 215. Another pre-existing mutation at position 453 in p6, Pro, was present at frequency 0.07 by day 6 p/s and increased to 0.71 at day 103 p/s, but then lost dominance at day 148 p/s, and balanced with initially dominant Ser at a later time point. The similarities between mutation patterns observed at positions 215, 389, and 453 in subject D-5018 are intriguing, and warrant further studies to reveal the underlying mechanism of the balancing of different amino acids. A dynamic interplay between viral quasispecies at position 370 provides evidence that a sequential escape and reversion in Gag is not a rare event during the first year of HIV-1 subtype C infection (Figure 3). A minor pre-existing Thr was observed at frequency 0.07 at day 6 p/s but accounted for 1.0 at day 103 p/s, demonstrating a successful complete escape from the wild type Ala. However the Thr frequency gradually declined and was completely substituted by the wild type-matching Ala by day 301 p/s, exemplifying a complete escape followed by a complete reversion to the wild type. Val, the *de novo* amino acid substitution at position 223 in p24 (Figure 3D), appeared at day 301 p/s and gradually reached 1.0 in the pool of viral quasispecies by day 483 p/s, suggesting a potential contribution of this mutation to viral fitness.

In contrast to subject D-5018, subject E-3430 dropped viral RNA load below 400 copies/ml within 30 days p/s, but also demonstrated increase of viral load accompanied by CD4+ T cells drop around 200 days p/s (Figure 4A). Subject E-3430 had a relatively high number of reverse mutations to HIV-1 subtype C wild type (Figure 4B), suggesting a viral transmission from a genetically distinct partner. Most reverse mutations to wild type in subject E-3430 occurred early and were pre-existing, resulting in a quick elimination of alternative amino acids from the pool of viral quasispecies (e.g., amino acid positions 42, 92, 141, 151, 298, 341, 411, 412, 414, 431, 443, 449, 459, and 462 in Figure 4B). The increase of viral RNA load around 200 days p/s was accompanied by escape mutations from wild type such as at amino acid positions 147 and 441 (Figure 4C), and escape mutation from non-wild type at position 28 (Figure 4D). Similarly to subject D-5018, the causality in the relationship between viral mutations and viral load changes in subject E-3430 should be taken cautiously, and might require support of immunologic analysis in future studies. At amino acid position 111 in p17, the *de novo* reversion to Ser appeared at frequency 0.10 at day 274 p/s on the background of interplay of two other amino acids, Cys and Gly (Figure 4B). However Ser was gone by day 305 p/s suggesting that the wild type-matching viral variant did not have a replication advantage in the host environment of subject E-3430. At position 441 in p1, the *de novo* His-to-Tyr substitution resulted in a complete escape from the wild type at days 243 p/s and 274 p/s, and was followed by minor reversion of His at days 305, 339, and 404 p/s, apparently suggesting immune suppression of the wild type variant. A mutation at position 147 in p24 demonstrated a fast Ile-to-Leu escape that appeared at day 150 p/s and reached completeness by day 274 p/s. There was a sequential escape-reversion-escape series at position 374 in p2 from day 274 p/s to day 404 p/s. An unusual double escape was observed at position 375 in p2 demonstrating a complex sequential escape(Lys)-escape(His)-reversion(Asn)-escape(Lys)-reversion(Asn) pattern. The *de novo* minor Lys at position 375 appeared at day 150 p/s, and was followed by the appearance of His as a dominant amino acid at frequency 0.67 at day 243 p/s, leaving Lys and Asn at frequency 0.17 each. However the spectrum of viral quasispecies changed to Asn at 0.60, Lys at 0.30, and His at 0.10 at day 274 p/s. The interplay between viral quasispecies resulted in a loss of His by day 305 p/s accompanied by sequential switching of Lys and Asn frequencies at later time points.

In subject F-3505, similarly to subject E-3430, viral RNA load dropped about 2.0 log_10_ within the first two months p/s, stayed relatively low, but then increased around 200 days p/s (Figure 5A). The increase in viral load coincided with a few reverse mutations to wild type at positions 114 and 302 (Figure 5B) and escape mutations from the wild type at amino acid positions 242 and 370 (Figure 5C). However functional immulogic studies are necessary to resolve causality in the relationship between viral mutations and viral load in subject F-3505. The reverse mutation at position 146 (Ser-to-Ala) at day 323 p/s was followed by a minor escape mutation (Pro at frequency 0.25) at day 447 p/s. Escape mutations from the wild type at positions 242, 252, and 370 gradually reached dominance and were completed by days 323, 447, and 323 p/s, respectively. Two sequential transient escape mutations from the wild type were evident at position 244. Ser appeared at frequency 0.17 at day 203 p/s but disappeared by day 261 p/s, while Ala appeared at frequency 0.14 at day 261 p/s and disappeared by day 323 p/s. Similar sequential transient escapes from the non-wild type occurred at position 182: Pro at frequency 0.13 was evident at day 98 p/s but gone at day 203 p/s, and was followed by Lys at 0.14 at day 261 p/s that also disappeared by day 323 p/s.

### Timing of Amino Acid Substitutions in Gag

To address the timing of Gag mutations in relation to estimated seroconversion, three analytical approaches were taken. First, the time of amino acid substitutions in Gag was analyzed cumulatively by type of mutation irrespective of the individual contribution by each subject, providing an overall summary of the time and timing differences between different types of mutations. Second, time to specific Gag mutations were analyzed by fitting the Cox proportional hazards models with frailties, which allowed for correlation among data points contributed by each subject. Lastly, the timing of reverse and escape mutations in Gag were analyzed within individuals.

Table 1 summarizes the timing of first appearance, dominance, completeness, and loss of mutations among two types of Gag mutations, reverse mutations and escape mutations from the wild type. The appearance of Gag mutations was analyzed overall and within subsets of minor, transient, dominant, and complete mutations. Overall reverse mutations appeared at earlier time points than escape mutations from the wild type. The median (IQR) of the first appearance of reverse mutations in Gag was 62 (44–139) days p/s, while the median time of appearance of escape mutations from the wild type was 234 (169–326) days p/s (p<0.001). The time of appearance of minor mutations was 312 (160–418) days p/s and 349 (240–415) days p/s for reverse and escape mutations, respectively (p = 0.031), while appearance of transient mutations did not differ significantly between reverse mutations and escape mutations from the wild type. Among Gag mutations that reached dominance, time to appearance of reverse mutations (median (IQR) of 54 (30–78) days p/s) was significantly earlier than the appearance of escape mutations from the wild type (median (IQR) of 104 (47–198) days p/s, (p<0.001). Similar timing relationships between reverse and escape mutations were found for the appearance of Gag mutations that became complete. The median (IQR) appearance of reverse mutations that reached completeness was 54 (30–78) days p/s, whereas the median appearance of escape mutations that became complete later was 90 (44–196) days p/s (p = 0.006). Time until reaching dominance was significantly shorter for reverse mutations (median (IQR) of 58 (44–105) days p/s) than for escape mutations from the wild type (median (IQR) of 219 (90–326) days p/s, p<0.001). A similar pattern was evident for mutations in Gag that reached completeness. The medians (IRQ) of completeness were 152 (100–176) days p/s and 243 (101–370) days p/s for reverse mutations and escape mutations from the wild type, respectively (p = 0.001). Therefore, based on multiple comparisons, we conclude that reverse mutations in Gag occur significantly earlier than escape mutations from the wild type.

**Table 1:** Timing of reverse mutations and escape mutations from the wild type in Gag during primary HIV-1 subtype C infection.

	Reverse mutations			Escape mutations from the wild type			p-value* for timing comparison
	n	Time, days p/s, median (IQR)		n	Time, days p/s, median (IQR)		
**Time of Appearance:** all mutations	353	62	(44; 139)	738	234	(169; 326)	**<0.001**
Minor mutations (did not reach dominance and were not lost)	57	312	(160; 418)	233	349	(240; 415)	**0.031**
Transient minor mutations (did not reach dominance and were lost by 500 days p/s)	39	196	(116; 252)	438	219	(152; 261)	0.250
Mutations that reached dominance	257	54	(30; 78)	67	104	(47; 198)	**<0.001**
Mutations that reached completeness (fixed)	209	54	(30; 78)	39	90	(44; 196)	**0.006**
**Time of Dominance:** mutations that reached dominance	257	58	(44; 105)	67	219	(90; 326)	**<0.001**
**Time of Completeness:** mutations that reached completeness (fixed)	209	152	(100; 176)	39	243	(101; 370)	**0.001**

*Mann-Whitney Rank Sum Test.

The number of escape mutations from non-wild type was too small for analysis within subsets, and therefore only major groups were included in the comparative analysis. The time of first appearance of escape mutations from the non-wild type (median (IQR) of 188 (103–309) days p/s) was later than that of reverse mutations (p<0.001) but earlier than that of escape mutations from the wild type (p<0.001). The time of reaching dominance for escape mutations from non-wild type (median (IQR) of 230 (150–389) days p/s) was later than that for reverse mutations (p<0.001), but did not significantly differ from the time to dominance for escape mutations from the wild type (p = 0.14). Time to completeness for escape mutations from non-wild type (median (IQR) of 243 (211–427) days p/s) was significantly longer than for reverse mutations (p = 0.001) but did not differ from escape mutations from the wild type (p = 0.27).

To address whether timing of Gag mutations is associated with their presence in the pool of viral quasispecies at the earliest time point, we analyzed the timing of reverse and escape mutations by whether the mutations were pre-existing or *de novo* mutations (Table 2). We note that most reverse mutations were pre-existing while most escape mutations evolved *de novo*. The time of appearance of the total reverse mutations and escape mutations from the wild type did not significantly differ within subset of the *de novo* mutations (p = 0.16), but was shorter for pre-existing reverse (p = 0.044) mutations in Gag. For the *de novo* mutations, appearance of reverse mutations that reached dominance (median of 113 days) occurred significantly earlier than appearance of escape mutations from the wild type (median of 174 days, p = 0.041). In contrast, for the pre-existing mutations, the appearance of reverse mutations that reached dominance (median of 52 days) occurred slightly later than appearance of escape mutations from the wild type (median of 44 days) but did not reach statistical significance (p = 0.072). Similar patterns were found for appearance of reverse and escape mutations that reached completeness (Table 2; p = 103 and p = 0.022 for *de novo* and pre-existing mutations, respectively). Time until reaching dominance or time to completeness did not differ between reverse and escape mutations within either *de novo* or pre-existing mutations in Gag.

**Table 2:** *De novo* and pre-existing reverse mutations and escape mutations from the wild type in Gag during primary HIV-1 subtype C infection.

	Reverse mutations			Escape mutations from the wild type			p-value* for timing comparison
	n	Time, days p/s, median (IQR)		n	Time, days p/s, median (IQR)		
**Time of Appearance:**							
*De novo* mutations	113	223	(137; 345)	696	235	(181; 326)	0.159
Pre-existing mutations	240	52	(30; 78)	42	65	(44; 226)	**0.044**
Mutations that reached dominance:							
*De novo* mutations	30	113	(58; 205)	42	174	(105; 233)	**0.041**
Pre-existing mutations	227	52	(28; 65)	26	47	(7; 52)	0.072
Mutations that reached completeness (fixed):							
*De novo* mutations	17	139	(46; 184)	23	170	(94; 203)	0.103
Pre-existing mutations	193	52	(30; 65)	17	46	(7; 50)	**0.022**
**Time of Dominance:**							
*De novo* mutations	33	223	(117; 264)	43	261	(2072; 349)	0.074
Pre-existing mutations	227	58	(30; 103)	26	92	(44; 177)	0.104
Mutations that reached completeness (fixed):							
*De novo* mutations	17	139	(55; 230)	23	240	(113; 260)	0.092
Pre-existing mutations	193	58	(30; 79)	16	48	(26; 92)	0.608
**Time of Completeness:**							
* De novo* mutations	17	204	(139; 241)	23	323	(228; 377)	0.052
Pre-existing mutations	193	152	(100; 174)	17	139	(100; 294)	0.365

*Mann-Whitney Rank Sum Test.

Taking into account the correlation among an individual subject's values, we fit a Cox proportional hazards models with frailties to compare time to first appearance (dominance, or completeness) for reverse mutations and escape mutations from the wild type. The number of escape mutations from non-wild type was small, and therefore not included in the comparative analysis. Time of first appearance of reverse mutations occurred significantly earlier than for escape mutations from the wild type (hazard ratio (HR): 2.6; 95% CI: 2.3–3.0). Also, reverse mutations took significantly less time to reach dominance (and completeness) as compared to escape mutations from the wild type (for dominance: HR 4.8 (3.4–6.8)); for completeness: HR 3.6 (2.3–5.5)). These analyses provide further evidence that reverse mutations in Gag take place at earlier times than do escape mutations from the wild type.

The number of reverse mutations per subject (median (IQR): 7 (4–13)) was smaller than the number of escape mutations from the wild type (median (IQR): 16 (12–23); p<0.001). Reverse mutations appeared in Gag significantly earlier than did escape mutations from the wild type in 22 of 42 (52.4%) comparable subjects. In the remaining 20 cases, there was no significant difference between times of appearance. The median (IQR) time of first appearance of reverse mutations was 58 (44–156) days, while the time of first appearance of escape mutations from the wild type was 236 (198–261) days, providing further support for the identified timing pattern in the evolution of different types of Gag mutations. Fewer comparisons were available for the intra-patient analysis of time to dominance and completeness because most escape mutations from the wild type never exceeded the level of 50% in the pool of viral quasispecies. Shorter time to dominance for reverse mutations was observed in 6 of 23 (26%) comparable subjects. The time to dominance of reverse mutations (median (IQR) of 58 (29–152) days) was significantly shorter than that of escape mutations from the wild type (median (IRQ) of 197 (136–286) days, p<0.001). Time to completeness for reverse mutations (median (IRQ) of 146 (102–204) days) was significantly shorter than that for escape mutations from the wild type (median (IRQ) of 244 (124–329) days, p = 0.018).

### Timing of Mutations across Gag Cleavage Products

**Figure 6:**
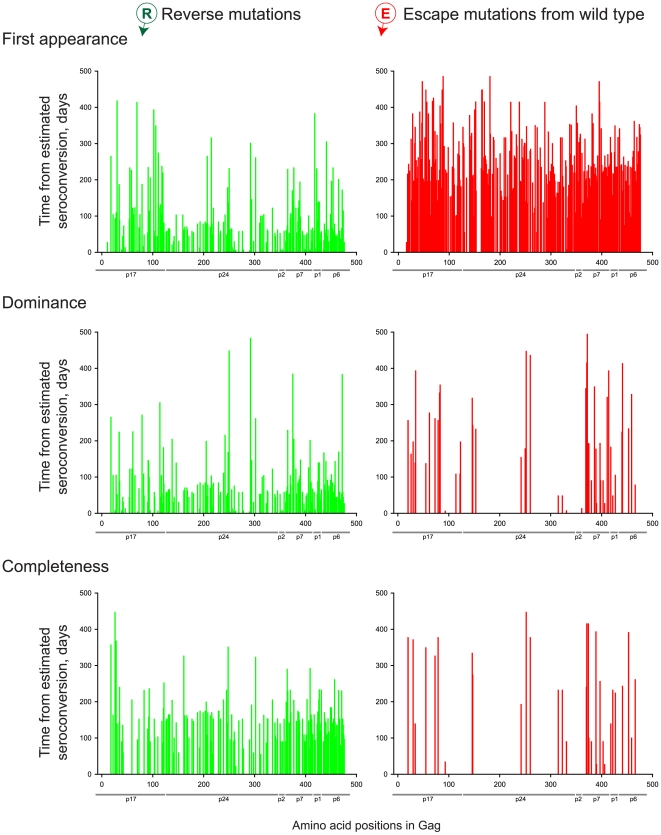
Timing of *in vivo* Gag mutations by the times of appearance, dominance and completeness. Reverse mutations to the wild type are compared to escape mutations from the wild type.

We assessed whether the timing of Gag mutations differs between Gag cleavage products by performing analyses within p17, p24, and p2p7p1p6 (Tables 3, 4, 5). In all analyzed regions, reverse mutations appeared faster than escape mutations from the wild type (Figure 6). However, the magnitude of association between time to first appearance and mutation type varied according to the Gag cleavage product in which the mutations occurred (interaction test, p = 0.008), as did the association between time to completeness and mutation type (interaction test, p = 0.02). In contrast, the association between time to dominance and mutation type did not vary significantly across Gag cleavage products (interaction test, p = 0.2); neither did the association between time to completeness and mutation type (interaction test, p = 0.2).

**Table 3:** Time to 1^st^ appearance of amino acid substitutions by Gag cleavage product.

	Gag mutations				
	Reverse		Escape from wild type		
Gag cleavage product	n	Time, median (IQR)	n	Time, median (IQR)	HR, 95% CI
p17	92	88 (44–206)	171	227 (150–350)	2.1 (1.6–2.8)
p24	107	62 (44–102)	235	235 (180–325)	5.0 (3.7–6.6)
p2p7p1p6	154	62 (44–141)	332	235 (169–317)	2.8 (2.2–3.4)

**Table 4:** Time to Dominance of amino acid substitutions by Gag cleavage product.

	Gag mutations				
	Reverse		Escape from wild type		
Gag cleavage product	n	Time, median (IQR)	n	Time, median (IQR)	HR, 95% CI
p17	59	58 (44–164)	18	212 (138–311)	3.0 (1.5–6.0)
p24	92	62 (30–88)	16	237 (48–302)	7.3 (2.9–18.8)
p2p7p1p6	109	62 (44–122)	35	219 (90–334)	5.6 (3.3–9.7)

**Table 5:** Time to Completeness of amino acid substitutions by Gag cleavage product.

	Gag mutations				
	Reverse		Escape from wild type		
Gag cleavage product	n	Time, median (IQR)	n	Time, median (IQR)	HR, 95% CI
p17	36	152 (127–233)	9	349 (295–377)	2.2 (0.9–5.2)
p24	77	152 (95–170)	11	274 (170–329)	10.4 (3.0–36.2)
p2p7p1p6	97	147 (108–204)	20	228 (98–340)	3.0 (1.6–5.6)

## Discussion

Timing patterns of viral mutational pathways in the natural course of HIV-1 infection are not well understood, and denote a knowledge gap in HIV research. In this study, we addressed timing of *in vivo* Gag mutations during primary HIV-1 subtype C infection in a prospective cohort of 8 acutely and 34 recently infected individuals from Botswana. Despite a significant heterogeneity between subjects in the time of appearance, dominance, and completeness of amino acid substitutions in Gag during the early phase of HIV-1 subtype C infection, we found that the *in vivo* reverse mutations to the wild type in Gag occurred significantly earlier than escape mutations from the wild type.

Our results show that the appearance, dominance, and completeness of reverse mutations in Gag occurs significantly sooner than escape mutations from the wild type, which is consistent with a recent report [2]. It is believed that reverse mutations restore the wild type viral sequence, and apparently account for more fit virus with higher replication potential. However the question “When do viral mutations occur?” has not been addressed by previous studies. Our finding that reverse mutation in Gag appears at a median of 62 days following seroconversion may have important public health implications, as it may suggest the existence of a “window of opportunity” for early interventions of about two months after seroconversion.

Early initiation of antiretroviral therapy in those subjects who maintain high viral load might slow down viral reversions by reducing viral replication. Our results provide a rationale for testing a hypothesis that early therapeutic vaccination (e.g., within two months p/s) may prevent reverse mutations and reduce viral set point. The amino acid positions that undergo frequent reversions represent critical targets, and the time before the reverse mutations occur denotes the opportunity to prevent these mutations. If the vaccine can abrogate or at least slow down reverse mutations, the level of viral replication could be reduced, allowing more time for the immune system to regain adequate immune response.

The current study generates a number of other hypotheses that can be addressed in future studies. It would be important to determine relationships between observed Gag mutations and virus-specific CD8+ and CD4+ T cell responses. Timing of virus-specific T cell responses is critical for understanding whether immune responses drive or follow viral mutations. It would be crucial to determine whether ART affects the timing of viral mutational pathways in Gag, and if so, the extent of ART contribution. Knowledge of timing patterns of co-variation and compensatory mutations in Gag could further facilitate our understanding of viral evolution in the early phase of HIV-1 infection. In contrast to the cross-sectional studies, the current study focuses on multiple time points assuming that functionally relevant viral variants should be detectable in a set of longitudinal samples even with a relatively small number of viral sequences per time point. Conversely, if viral variants are not detectable in longitudinal sampling, its functional relevance may be low.

The results of our studies seem to resolve the discrepancy in understanding of relationships between HIV-1 reverse mutations to and escape mutations from the wild type sequence highlighted recently by Brumme & Walker [54]. Our data are congruent with recent studies [2], [17], [55], [56], and demonstrate that after seroconversion, the overall number of reverse mutations is smaller than the number of escape mutations, but reverse mutations occur earlier and are mostly dominant or complete, while escape mutations from the wild type occur later and most are transient.

In case of transmission of multiple or closely related viral variants, the selection of one viral population over time was not uncommon. The observed pattern suggested a transmission of more than a single viral variant, although we cannot exclude transmission of a single variant followed by early diversification in response to early immune responses as described recently by Goonetilleke et al. [17]. Irrespective of the origin of viral mutation, the presence of two (or multiple) amino acids at an early time point followed by a selection of one viral population is an important phenomenon because it shortens the time to reach dominance and completeness of the more fit virus. For example, we found that the time to a complete T242N escape in well-studied epitope TW10 [13], [52], [53], [57], [58] is shorter in subjects infected with a mix of Thr and Asn than in individuals infected with the wild type virus only (unpublished data).

The study identified timing of different types of *in vivo* Gag mutations in primary HIV-1 subtype C infection in relation to the estimated time of seroconversion by using a novel approach of direct mapping of Gag mutations along the timeline of HIV-1 infection. Overall, the *in vivo* reverse mutations to the wild type occurred significantly earlier than escape mutations from the wild type. This shorter time to incidence of reverse mutations remained in the subsets of *in vivo* Gag mutations that reached dominance or completeness.

## Supporting Information

Figure S1 Time points post-seroconversion (p/s) and the number of sequences. Patient ID and abbreviated code are shown in the first two columns, and are followed by the Fiebig stage of each subject. The distribution of 42 cases by Fiebig staging included 8 (19%) cases in stage II; 10 (24%) cases in stage IV; 18 (43%) cases in stage V; and 4 (10%) cases on the edge of stages V and VI (extremely faint p31 band evident for a transition from stage V to stage VI). According to the original paper [35] and application of Fiebig staging to HIV-1 subtype C samples,[59] the assumption was made that the beginning of Fiebig stage III coincides with the time of detectable seroconversion (time 0), and the mean duration of Fiebig stage III is 3 days, of stage IV is 6 days, and of stage V is 70 days. Based on these estimates, the time from seroconversion until detection was assumed to average 6 days for subjects in stage IV (3 days of phase III and 3 days to the mid-point of phase IV), 44 days for subjects in stage V (9 days of phases III and IV and 35 days to the mid-point of phase V), and 79 days for stage V/VI (9 days of phases III and IV, and 70 days of phase V). Time points from seroconversion are shown for each subjects as day post-seroconversion, p/s with corresponding number of gag sequences generated in the study. The color-coding scheme is presented at the top of the figure. Sequences obtained from viral RNA template are delineated by pink, sequences generated from proviral DNA template are shown without highlight, and the cumulative number of sequences acquired from both RNA and DNA template are highlighted by dark red. The total number of gag sequences generated in the study is shown at the bottom right.
(0.08 MB EPS)

Figure S2 Temporal relationships between frequency distributions of Gag quasispecies in acutely infected subject A-1811 at 10 time points from day 6 p/s to day 356 p/s. MHC class I HLA profile is presented at the top of the figure. Gray bars at the background of each graph delineate 100-days time intervals for better comparison between dynamics of viral load/CD4 (Figure S2.A), and time of Gag mutations (Figure S2.B to S2.D). Number with the letter at the top of each graph in B, C, and D represents amino acid position in Gag according to the HXB2 numbering (http://www.hiv.lanl.gov/) and the most frequent amino acid in the HIV-1 subtype C consensus. Green circled R with arrow delineates reverse mutation to wild type virus, and red circled E with arrow indicates escape mutations from wild type. A: Dynamics of plasma RNA load (red squares), proviral DNA load (green diamonds), and CD4+ T cell count (blue triangles) over the follow up period. Time of ART initiation is outlined by dashed arrow. B: Reverse mutations to HIV-1 subtype C wild type virus. C: Escape mutations from HIV-1 subtype C wild type virus. D: Escape mutations from non-wild type (wild type amino acid is not involved).
(1.95 MB EPS)

Figure S3 Temporal relationships between frequency distributions of Gag quasispecies in acutely infected subject B-2865 from day 10 p/s to day 223 p/s. See legend to Figure S2.
(1.62 MB EPS)

Figure S4 Temporal relationships between frequency distributions of Gag quasispecies in acutely infected subject C-3312 from day 4 p/s to day 445 p/s. See legend to Figure S2. No escape mutations from non-wild type were detected in subject C-3312 (no Figure S4.D is presented).
(1.69 MB EPS)

Figure S5 Temporal relationships between frequency distributions of Gag quasispecies in acutely infected subject G-3603 from day 4 p/s to day 345 p/s. See legend to Figure S2.
(1.50 MB EPS)

Figure S6 Temporal relationships between frequency distributions of Gag quasispecies in acutely infected subject H-5582 from day 16 p/s to day 347 p/s. See legend to Figure S2.
(1.46 MB EPS)

Figure S7 Alignment of translated amino acids in subject D-5018 at 7 time points from day 6 p/s to day 483 p/s. Sampling time of sequences is outlined in sequence name, and is shown in days p/s as a 3-digit number after the abbreviated patient code “D_”. For example, D_006_01 delineate sampling at day 6 p/s, sequence number 1, D_103_02 outlines sampling at day 103 p/s, sequence number 2, etc. Sequences originating from viral RNA template are delineated with “RNA” at the end of sequence name, while all other sequences were generated from proviral DNA template. Shown sequences are compared to the first sequence in alignment. Note that numbering above alignment represents sequences in subject D-5018, and does not correspond to Gag amino acid numbering of HXB2.
(0.11 MB EPS)

Figure S8 Alignment of translated amino acids in subject E-3430 at 8 time points from day 30 p/s to day 404 p/s. Sampling time of sequences is outlined in sequence name, and is shown in days p/s as a 3-digit number after the abbreviated patient code “E_”. For example, E_030_01 delineate sampling at day 30 p/s, sequence number 1, E_059_01 outlines sampling at day 59 p/s, sequence number 1, etc. Sequences originating from viral RNA template are delineated with “RNA” at the end of sequence name, while all other sequences were generated from proviral DNA template. Shown sequences are compared to the first sequence in alignment. Note that numbering above alignment represents sequences in subject E-3430, and does not correspond to Gag amino acid numbering of HXB2.
(0.12 MB EPS)
