# Supplementary material for: Timing Constraints of In Vivo Gag Mutations during Primary HIV-1 Subtype C Infection
Source: PLoS One. 2009 Nov 5;4(11):e7727. doi: 10.1371/journal.pone.0007727 (PMC2768328; doi:10.1371/journal.pone.0007727)
Supplement: Figure S1 — Time points post-seroconversion (p/s) and the number of sequences. Patient ID and abbreviated code are shown in the first two columns, and are followed by the Fiebig stage of each subject. The distribution of 42 cases by Fiebig staging included 8 (19%) cases in stage II; 10 (24%) cases in stage IV; 18 (43%) cases in stage V; and 4 (10%) cases on the edge of stages V and VI (extremely faint p31 band evident for a transition from stage V to stage VI). According to the original paper [35] and application of Fiebig staging to HIV-1 subtype C samples,[59] the assumption was made that the beginning of Fiebig stage III coincides with the time of detectable seroconversion (time 0), and the mean duration of Fiebig stage III is 3 days, of stage IV is 6 days, and of stage V is 70 days. Based on these estimates, the time from seroconversion until detection was assumed to average 6 days for subjects in stage IV (3 days of phase III and 3 days to the mid-point of phase IV), 44 days for subjects in stage V (9 days of phases III and IV and 35 days to the mid-point of phase V), and 79 days for stage V/VI (9 days of phases III and IV, and 70 days of phase V). Time points from seroconversion are shown for each subjects as day post-seroconversion, p/s with corresponding number of gag sequences generated in the study. The color-coding scheme is presented at the top of the figure. Sequences obtained from viral RNA template are delineated by pink, sequences generated from proviral DNA template are shown without highlight, and the cumulative number of sequences acquired from both RNA and DNA template are highlighted by dark red. The total number of gag sequences generated in the study is shown at the bottom right. (0.08 MB EPS) [file pone.0007727.s001.pdf]

Figure S1. Time points post seroconversion (p/s) and the number of sequences.

Color coding:

21 RNA sequences

21 DNA sequences

21 RNA + DNA sequences

Patient ID	Abbreviated Code	Fiebig stage	Timepoints:	1st	2nd	3rd	4th	5th	6th	7th	8th	9th	10th	Total number of sequences
B001729-5	NA	V-VI	days p/s	169	231	354	448							42
			sequences, n	9	9	8	16							
B001811-6	A	II	days p/s	6	22	27	55	83	97	111	147	266	356	73
			sequences, n	6	10	6	6	5	15	6	5	6	8	
B002381-4	OC	IV	days p/s	27	86	208	300	393	482					84
			sequences, n	16	15	10	18	19	6					
B002530-1	OE	V	days p/s	52	81	122	205	236						68
			sequences, n	16	15	17	7	13						
B002604-4	OG	V	days p/s	104	163	194	219	317						60
			sequences, n	18	8	14	12	8						
B002761-0	OJ	IV	days p/s	44	135	224	316							62
			sequences, n	16	18	19	9							
B002865-8	B	II	days p/s	10	18	74	103	164	223					72
			sequences, n	9	2	21	16	14	10					
B002869-2	OM	V-VI	days p/s	86	178									38
			sequences, n	21	17									
B002990-7	OQ	V	days p/s	52	139	233	320	415						44
			sequences, n	10	12	7	7	8						
B003079-8	OS	V	days p/s	62	153	243	326	411	494					73
			sequences, n	10	21	12	6	17	7					
B003091-0	OU	IV	days p/s	13	44	104	196	289	349	471				106
			sequences, n	10	13	10	36	10	17	10				
B003234-6	OW	IV	days p/s	13	105	224	288	382	469					67
			sequences, n	8	23	13	12	6	5					
B003251-8	OX	V	days p/s	58	152	245	341	407	464					50
			sequences, n	13	8	6	7	9	7					
B003244-1	OY	V	days p/s	51	143	233	328	391	423					23
			sequences, n	2	2	9	5	1	4					
B003285-7	OZ	V	days p/s	78	170	261	280	326	395					63
			sequences, n	17	20	8	9	7	2					
B003312-8	C	II	days p/s	4	95	109	201	292	325	445				143
			sequences, n	36	22	16	37	8	9	15				
B003354-0	O1	IV	days p/s	6	94	185	243	312						43
			sequences, n	10	7	12	8	6						
B003390-6	PA	V	days p/s	65	129	164	227							67
			sequences, n	30	18	10	9							
B003430-0	E	II	days p/s	30	59	150	243	274	305	339	404			99
			sequences, n	24	10	9	6	10	10	8	22			
B003481-1	PC	V	days p/s	54	146	236	333	388	418					80
			sequences, n	28	10	6	10	17	9					
B003505-9	F	II	days p/s	7	70	98	203	261	323	447				45
			sequences, n	7	1	8	6	7	4	12				
B003508-7	PD	V	days p/s	59	150	242	334	428						44
			sequences, n	9	10	9	9	7						
B003603-1	G	II	days p/s	4	95	197	295	345						70
			sequences, n	10	9	21	6	24						
B004872-1	PK	V	days p/s	47	108	135	195							40
			sequences, n	7	17	7	9							
B005018-8	D	II	days p/s	6	103	148	177	301	393	483				82
			sequences, n	29	7	7	9	11	11	8				
B005062-7	PO	IV	days p/s	6	34	97	193	277						44
			sequences, n	10	10	5	11	8						
B005065-5	PP	V	days p/s	44	100	226								31
			sequences, n	10	9	12								
B005099-4	QA	IV	days p/s	6	181	389								30
			sequences, n	11	8	11								
B005340-7	QC	V	days p/s	80	235	415								38
			sequences, n	10	21	7								

Figure S1 (continuing). Time points post seroconversion (p/s) and the number of sequences.

Patient ID	Abbreviated Code	Fiebig stage	Timepoints:	1st	2nd	3rd	4th	5th	6th	7th	8th	9th	10th	Total number of sequences
B005355-2	QD	V-VI	days p/s	79	199	255	420							59
			sequences, n	11	15	24	9							
B005511-6	QG	V	days p/s	48	229	415								6
			sequences, n	1	2	3								
B005582-7	H	II	days p/s	16	55	98	202	271	347					88
			sequences, n	9	20	19	19	7	14					
B005715-8	QI	IV	days p/s	20	204	322	384	476						44
			sequences, n	10	12	12	1	9						
B005768-1	QJ	IV	days p/s	8	71	198	256	344	377	471				77
			sequences, n	4	14	12	20	10	2	15				
B005849-1	QM	V	days p/s	48	239	357	415							21
			sequences, n	6	2	6	7							
B005867-9	QP	V	days p/s	48	232	353	414							42
			sequences, n	10	11	8	13							
B005943-9	QR	IV	days p/s	7	90	219	316	485						58
			sequences, n	9	6	13	14	16						
B006020-8	QS	V	days p/s	44	169	232	292	351						31
			sequences, n	8	5	3	4	11						
B006024-2	QT	V	days p/s	44	137	228	289	349						64
			sequences, n	11	11	13	18	11						
B006029-2	QU	V-VI	days p/s	84	114	174	266							43
			sequences, n	11	14	8	10							
B006343-8	RA	V	days p/s	54	142	240	328	389	422					84
			sequences, n	10	12	8	22	15	17					
B006380-0	RB	V	days p/s	52	142	240	332							40
			sequences, n	7	9	11	13							
													Total:	2,438
