# Supplementary material for: Timing Constraints of In Vivo Gag Mutations during Primary HIV-1 Subtype C Infection
Source: PLoS One. 2009 Nov 5;4(11):e7727. doi: 10.1371/journal.pone.0007727 (PMC2768328; doi:10.1371/journal.pone.0007727)
Supplement: Figure S7 — Alignment of translated amino acids in subject D-5018 at 7 time points from day 6 p/s to day 483 p/s. Sampling time of sequences is outlined in sequence name, and is shown in days p/s as a 3-digit number after the abbreviated patient code “D_”. For example, D_006_01 delineate sampling at day 6 p/s, sequence number 1, D_103_02 outlines sampling at day 103 p/s, sequence number 2, etc. Sequences originating from viral RNA template are delineated with “RNA” at the end of sequence name, while all other sequences were generated from proviral DNA template. Shown sequences are compared to the first sequence in alignment. Note that numbering above alignment represents sequences in subject D-5018, and does not correspond to Gag amino acid numbering of HXB2. (0.11 MB EPS) [file pone.0007727.s007.pdf]

Fig. S7. Subject D-5018. Alignment of translated amino acids.

```
                      10        20        30        40        50        60        70        80        90       100
              ....|....|....|....|....|....|....|....|....|....|....|....|....|....|....|....|....|....|....|....|
D_006_01      KIRLRPGGKKQYRLKHIVWASRELERFALNPGLLETSEGCKQIMKQLQPALQTGTEELRSLFNTVATLYCVHEKIEVRDTKEALDKIEEEQNKCQQKIQQ
D_006_01RNA   ....................................................................................................
D_006_02      ....................................................................................................
D_006_03      ....................................................................................................
D_006_03RNA   ....................................................................................................
D_006_04      ....................................................................................................
D_006_04RNA   ....................................................................................................
D_006_05      ....................................................................................................
D_006_05RNA   ....................................................................................................
D_006_06      ....................................................................................................
D_006_06RNA   ....................................................................................................
D_006_07      ....................................................................................................
D_006_07RNA   ....................................................................................................
D_006_08      ....................................................................................................
D_006_08RNA   ....................................................................................................
D_006_09      ....................................................................................................
D_006_09RNA   ....................................................................................................
D_006_10      ....................................................................................................
D_006_10RNA   ....................................................................................................
D_006_11      ....................................................................................................
D_006_11RNA   ....................................................................................................
D_006_12      ....................................................................................................
D_006_12RNA   ....................................................................................................
D_006_13RNA   ....................................................................................................
D_006_14RNA   ........................................................................K...........................
D_006_15RNA   ....................................................................................................
D_006_18RNA   ....................................................................................................
D_006_19RNA   ....................................................................................................
D_006_20RNA   ............................................................................................R.......
D_103_02      ....................................................................................................
D_103_03      ....................................................................................................
D_103_04      ....................................................................................................
D_103_05      ....................................................................................................
D_103_06      ....................................................................................................
D_103_08      ....................................................................................................
D_103_09      ....................................................................................................
D_148_01      ....................................................................................................
D_148_03      ....................................................................................................
D_148_04      ....................................................................................................
D_148_07      .................I..................................................................................
D_148_08      .................I..................................................................................
D_148_09      .................I..................................................................................
D_148_10      ....................................................................................................
D_177_01      ....................................................................................................
```

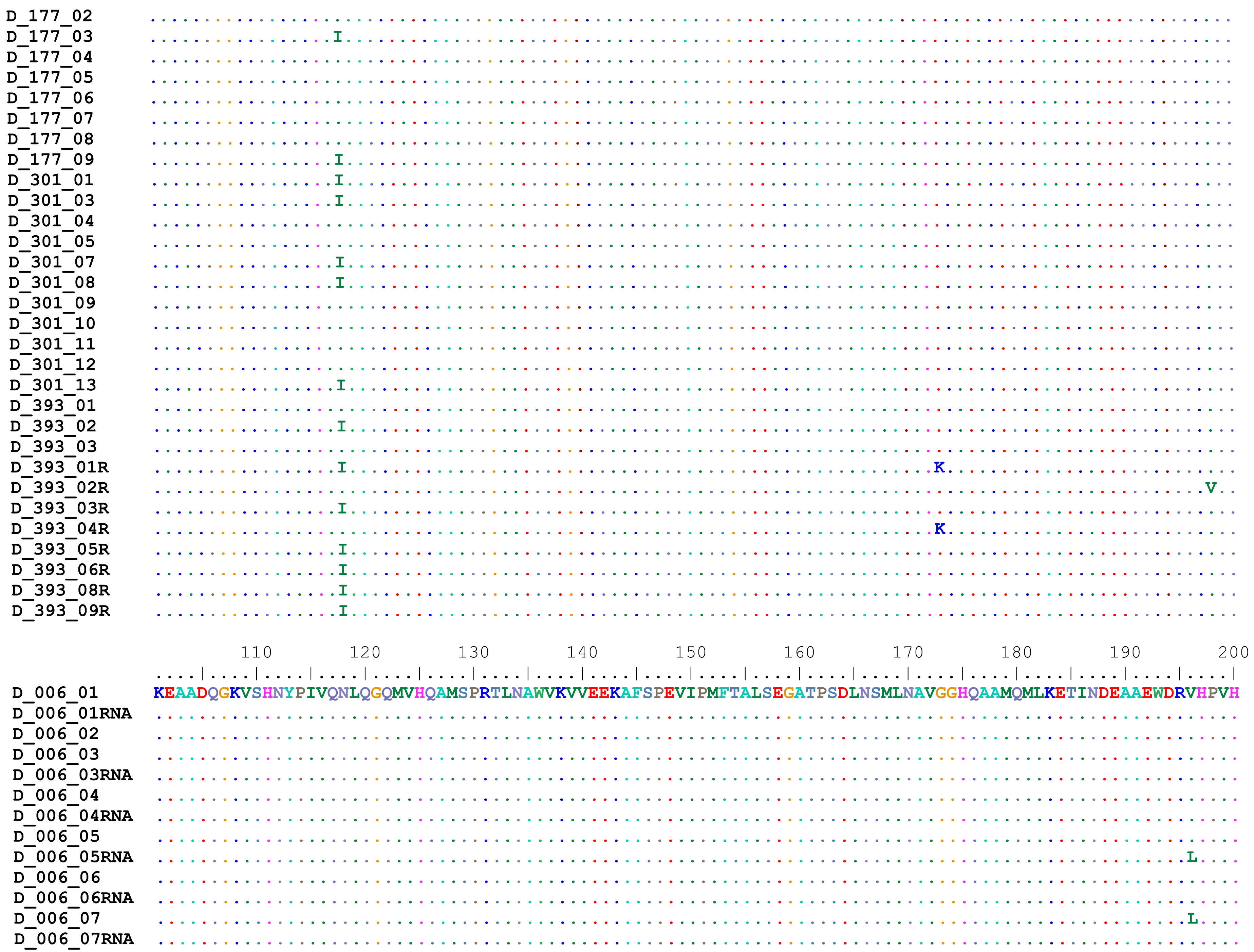
D_177_02
D_177_03 I
D_177_04
D_177_05
D_177_06
D_177_07
D_177_08
D_177_09 I
D_301_01 I
D_301_03 I
D_301_04
D_301_05
D_301_07 I
D_301_08 I
D_301_09
D_301_10
D_301_11
D_301_12
D_301_13 I
D_393_01
D_393_02 I
D_393_03
D_393_01R I K
D_393_02R V
D_393_03R I
D_393_04R K
D_393_05R I
D_393_06R I
D_393_08R I
D_393_09R I
110 120 130 140 150 160 170 180 190 200
D_006_01 KEAADQGKVSHNYPIVQNLQGQMVHQAMSPRTLNAWVKVVEEKAFSPEVIPMFTALSEGATPSDLNSMLNAVGGHQAAMQMLKETINDEAAEWDRVHPVH
D_006_01RNA
D_006_02
D_006_03
D_006_03RNA
D_006_04
D_006_04RNA
D_006_05
D_006_05RNA L
D_006_06
D_006_06RNA
D_006_07 L
D_006_07RNA

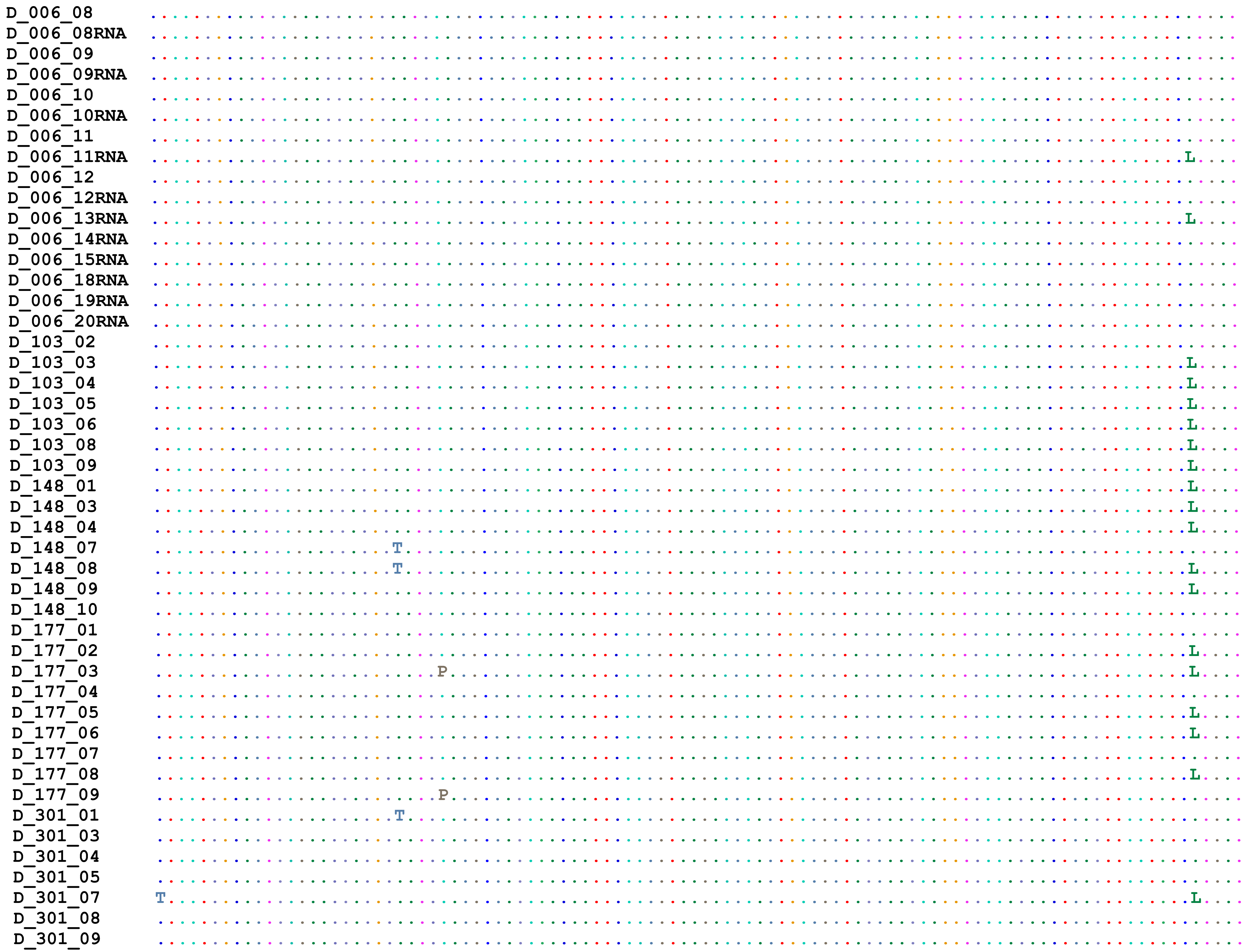
D_006_08
D_006_08RNA
D_006_09
D_006_09RNA
D_006_10
D_006_10RNA
D_006_11
D_006_11RNA L
D_006_12
D_006_12RNA
D_006_13RNA L
D_006_14RNA
D_006_15RNA
D_006_18RNA
D_006_19RNA
D_006_20RNA
D_103_02
D_103_03 L
D_103_04 L
D_103_05 L
D_103_06 L
D_103_08 L
D_103_09 L
D_148_01 L
D_148_03 L
D_148_04 L
D_148_07 T
D_148_08 T L
D_148_09 L
D_148_10
D_177_01
D_177_02 L
D_177_03 P L
D_177_04
D_177_05 L
D_177_06 L
D_177_07
D_177_08 L
D_177_09 P
D_301_01 T
D_301_03
D_301_04
D_301_05
D_301_07 T L
D_301_08
D_301_09

```
D_301_10        .............................................................................................L....
D_301_11        .............................................................................................L....
D_301_12        ..........................P..................................................................L....
D_301_13        ..........................P..................................................................L....
D_393_01        ....................................................................................................
D_393_02        ..........................P..................................................................L....
D_393_03        ....................................................................................................
D_393_01R       .....................T............................................C..........................L....
D_393_02R       ..........................P.........................................................................
D_393_03R       ..........................P..................................................................L....
D_393_04R       ..........................P.........................................................................
D_393_05R       ..........................P.........................................................................
D_393_06R       ..........................P.........................................................................
D_393_08R       ..........................P.........................................S........................L....
D_393_09R       ..........................P.......................................C..........................L....

                         210       220       230       240       250       260       270       280       290       300
                ....|....|....|....|....|....|....|....|....|....|....|....|....|....|....|....|....|....|....|....|
D_006_01        AGPAAPGQMREPRGSDIAGTTSTLQEQIAWMTSNPPIPVGDIYKRWIVLGLNKIVRMYSPVSILDIKQGPKESFRDYVDRFFKTLRAEQSTQEVKNWMTD
D_006_01RNA     ....................................................................................................
D_006_02        ....................................................................................................
D_006_03        ....................................................................................................
D_006_03RNA     ....................................................................................................
D_006_04        ....................................................................................................
D_006_04RNA     ....................................................................................................
D_006_05        ....................................................................................................
D_006_05RNA     ....................................................................................................
D_006_06        ....................................................................................................
D_006_06RNA     ....................................................................................................
D_006_07        ....................................................................................................
D_006_07RNA     ....................................................................................................
D_006_08        ....................................................................................................
D_006_08RNA     ....................................................................................................
D_006_09        ....................................................................................................
D_006_09RNA     ....................................................................................................
D_006_10        ....................................................................................................
D_006_10RNA     ....................................................................................................
D_006_11        ....................................................................................................
D_006_11RNA     ....................................................................................................
D_006_12        ....................................................................................................
D_006_12RNA     ....................................................................................................
D_006_13RNA     ....................................................................................................
D_006_14RNA     ....................................................................................................
D_006_15RNA     ....................................................................................................
D_006_18RNA     ....................................................................................................
D_006_19RNA     ....................................................................................................
```

```
D_006_20RNA  ....................................................................................................
D_103_02     ....................................................................................................
D_103_03     ....................................................................................................
D_103_04     ....................................................................................................
D_103_05     ....................................................................................................
D_103_06     ....................................................................................................
D_103_08     ....................................................................................................
D_103_09     ....................................................................................................
D_148_01     ....................................................................................................
D_148_03     ....................................................................................................
D_148_04     ....................................................................................................
D_148_07     ....................................................................................................
D_148_08     ....................................................................................................
D_148_09     ....................................................................................................
D_148_10     ....................................................................................................
D_177_01     ....................................................................................................
D_177_02     ....................................................................................................
D_177_03     ....................................................................................................
D_177_04     ....................................................................................................
D_177_05     ....................................................................................................
D_177_06     ....................................................................................................
D_177_07     ....................................................................................................
D_177_08     ....................................................................................................
D_177_09     ....................................................................................................
D_301_01     ....................................................................................................
D_301_03     ....................................................................................................
D_301_04     ....................................................................................................
D_301_05     ....................................................................................................
D_301_07     ....................................................................................................
D_301_08     ........................................................................P...........................
D_301_09     ....................................................................................................
D_301_10     ............................P.......................................................................
D_301_11     ....................................................................................................
D_301_12     ...V................................................................................................
D_301_13     ...V................................................................................................
D_393_01     ....................................................................................................
D_393_02     ....................................................................................................
D_393_03     ....................................................................................................
D_393_01R    ...V................................................................................................
D_393_02R    ....................................................................................................
D_393_03R    ...V................................................................................................
D_393_04R    ...V....................................................................P...........................
D_393_05R    ...V....................................................................P...........................
D_393_06R    ...V................................................................................................
D_393_08R    ....................................................................................................
D_393_09R    ....................................................................................................
```

```
                       310       320       330       340       350       360       370       380       390       400
              ....|....|....|....|....|....|....|....|....|....|....|....|....|....|....|....|....|....|....|....|
D_006_01      TLLVQNANPDCKTILRALGPGATLEEMMTACQGVGGPSHKARVLAEAMSQANNPNIMIQRGNFKGPRRIVKCFNCGKEGHIARNCRAPRKKGCWKCGKEG
D_006_01RNA   ....................................................................T...............................
D_006_02      ....................................................................T...............................
D_006_03      ....................................................................T...............................
D_006_03RNA   ....................................................................T...............................
D_006_04      ....................................................................T...............................
D_006_04RNA   ....................................................................T...............................
D_006_05      ....................................................................T...............................
D_006_05RNA   ..................................................T..A..............................................
D_006_06      ....................................................................T...............................
D_006_06RNA   ....................................................................T...............................
D_006_07      ....................................................................................................
D_006_07RNA   ....................................................................T...............................
D_006_08      ....................................................................T...............................
D_006_08RNA   ....................................................................T...............................
D_006_09      ....................................................................T...............................
D_006_09RNA   ....................................................................T...............................
D_006_10      ....................................................................T...............................
D_006_10RNA   ....................................................................T...............................
D_006_11      ....................................................................T...............................
D_006_11RNA   ..................................................T..A..............................................
D_006_12      ....................................................................T...............................
D_006_12RNA   ....................................................................T...............................
D_006_13RNA   ....................................................................................................
D_006_14RNA   ....................................................................T...............................
D_006_15RNA   ....................................................................................................
D_006_18RNA   ....................................................................T...............................
D_006_19RNA   ....................................................................T...............................
D_006_20RNA   ....................................................................T...............................
D_103_02      ..................................................T..A..............................................
D_103_03      ..................................................T.................T...............................
D_103_04      ..................................................T..A..............T...............................
D_103_05      ..................................................T.................................................
D_103_06      ..................................................T.................................................
D_103_08      ..................................................T..A..............................................
D_103_09      ..................................................T.................................................
D_148_01      ..................................................T.................................................
D_148_03      ....................................................................................................
D_148_04      ..................................................T..A..............................................
D_148_07      ....................................................................................................
D_148_08      ..................................................T.................................................
D_148_09      ....................................................................T...............................
D_148_10      ....................................................................T...............................
```

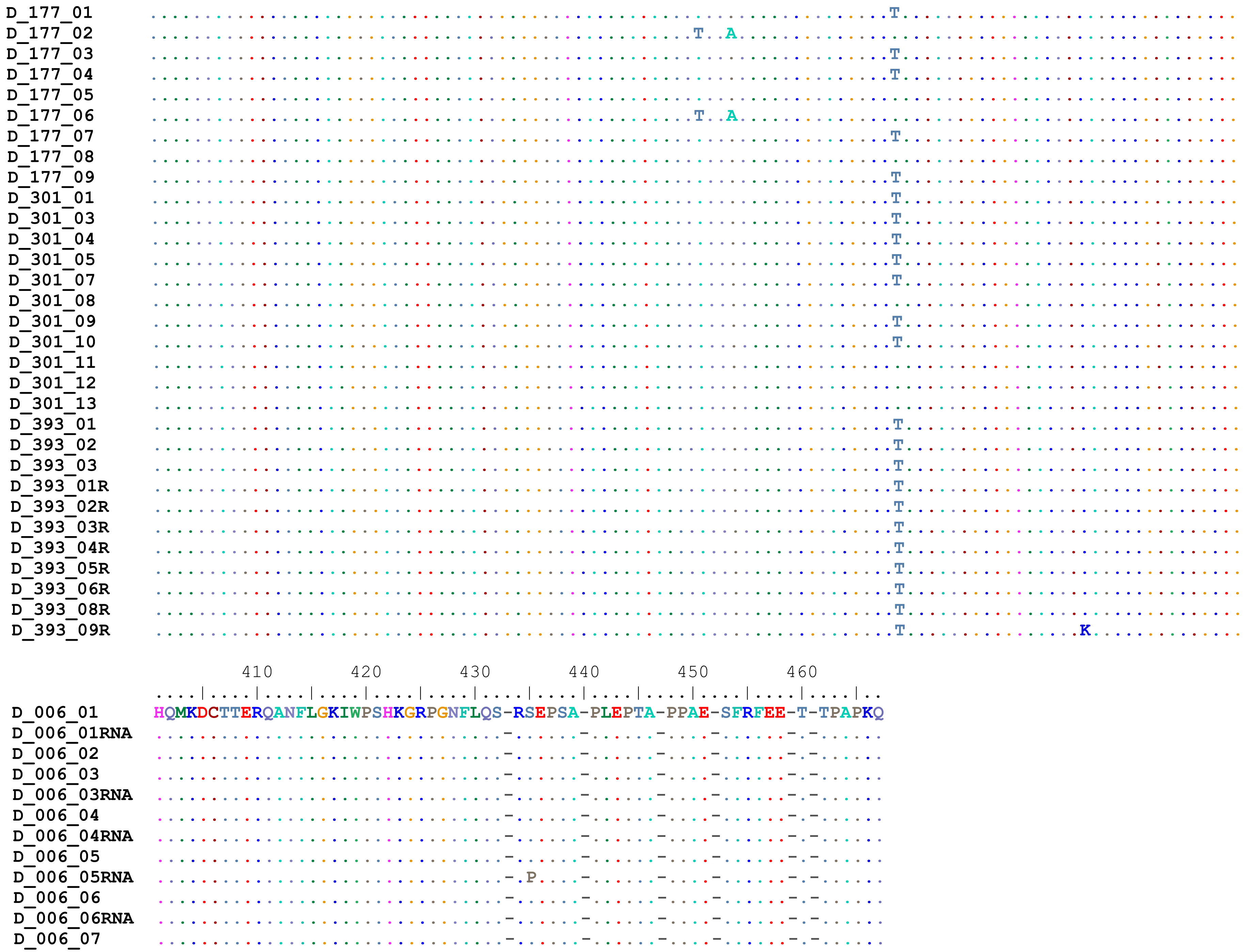

D_177_01
D_177_02
D_177_03
D_177_04
D_177_05
D_177_06
D_177_07
D_177_08
D_177_09
D_301_01
D_301_03
D_301_04
D_301_05
D_301_07
D_301_08
D_301_09
D_301_10
D_301_11
D_301_12
D_301_13
D_393_01
D_393_02
D_393_03
D_393_01R
D_393_02R
D_393_03R
D_393_04R
D_393_05R
D_393_06R
D_393_08R
D_393_09R
410
420
430
440
450
460
D_006_01 HQMKDCTTERQANFLGKIWPSHKGRPGNFLQS-RSEPSA-PLEPTA-PPAE-SFRFEE-T-TPAPKQ
D_006_01RNA
D_006_02
D_006_03
D_006_03RNA
D_006_04
D_006_04RNA
D_006_05
D_006_05RNA
D_006_06
D_006_06RNA
D_006_07

```
D_006_07RNA   ................................-......-......-....-......-.-......
D_006_08      ................................-......-......-....-......-.-......
D_006_08RNA   ................................-......-......-....-......-.-......
D_006_09      ................................-......-......-....-......-.-......
D_006_09RNA   ................................-......-......-....-......-.-......
D_006_10      ................................-......-......-....-......-.-......
D_006_10RNA   ................................-......-......-....-......-.-......
D_006_11      ................................-......-......-....-......-.-......
D_006_11RNA   ................................-.P....-......-....-......-.-......
D_006_12      ................................-......-......-....-......-.-......
D_006_12RNA   ................................-......-......-....-......-.-......
D_006_13RNA   ................................-......-......-....-......-.-......
D_006_14RNA   ................................-......-......-....-......-.-......
D_006_15RNA   ................................-......-......-....-......-.-......
D_006_18RNA   ................................-......-......-....-......-.-......
D_006_19RNA   ................................-......-......-....-......-.-......
D_006_20RNA   ................................-......-......-....-......-.-......
D_103_02      ................................-.P....-......-....-......-.-......
D_103_03      ........K.......................-.P....-......-....-......-.-......
D_103_04      ................................-.P....-......-....-......-.-......
D_103_05      ................................-.P....-......-....-......-.-......
D_103_06      ................................-......-......-....-......-.-......
D_103_08      ................................-.P....-......-....-......-.-......
D_103_09      ................................-......-......-....-......-.-......
D_148_01      ................................-......-......-....-......-.-......
D_148_03      ................................-......-......-....-......-.-......
D_148_04      ................................-......-......-..S.-......-.-......
D_148_07      ........K.......................-.P....-......-....-......-.-..T...
D_148_08      ................................-......-......-....-......-.-......
D_148_09      ................................-.P....-......-....-......-.-......
D_148_10      ................................-......-......-....-......-.-......
D_177_01      ................................-......-......-....-......-.-......
D_177_02      ................................-......-......-..S.-......-.-......
D_177_03      ................................-.P....-......-....-......-.-......
D_177_04      ................................-......-......-....-......-.-......
D_177_05      ................................-......-......-....-......-.-......
D_177_06      ................................-.P....-......-....-......-.-......
D_177_07      ................................-......-......-....-......-.-......
D_177_08      ................................-.P....-......-....-......-.-......
D_177_09      ................................-.P....-......-....-......-.-......
D_301_01      ................................-......-......-....-......-.-......
D_301_03      ................................-......-......-....-......-.-......
D_301_04      ................................-......-......-....-......-.-......
D_301_05      ................................-......-......-....-......-.-......
D_301_07      ................................-KP....-......-....-......-.-......
D_301_08      ................................-.P....-......-....-......-.-......
```

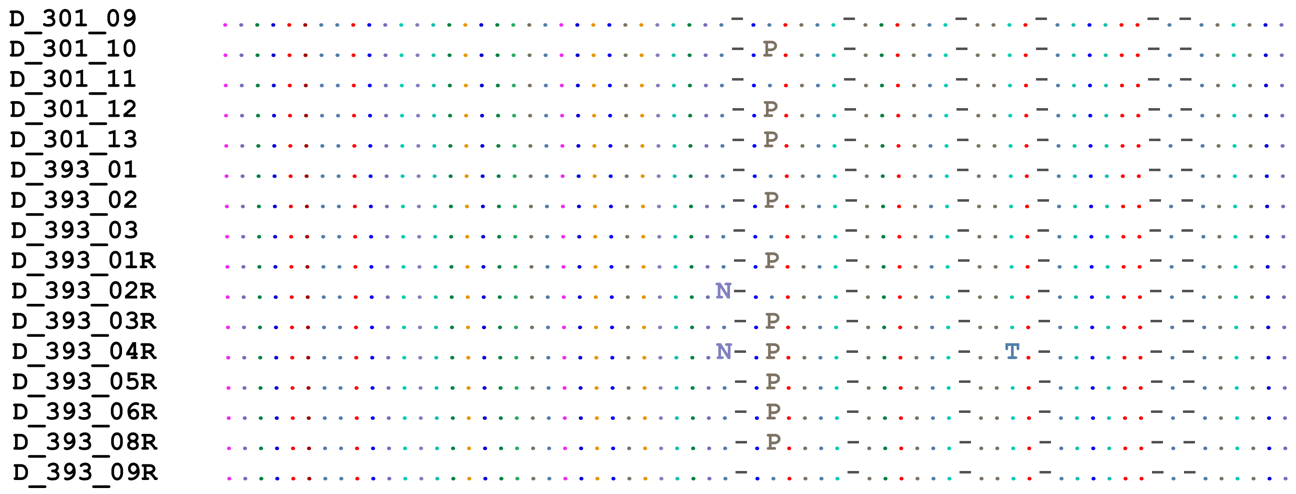
D_301_09
D_301_10
D_301_11
D_301_12
D_301_13
D_393_01
D_393_02
D_393_03
D_393_01R
D_393_02R
D_393_03R
D_393_04R
D_393_05R
D_393_06R
D_393_08R
D_393_09R
