# Supplementary material for: Timing Constraints of In Vivo Gag Mutations during Primary HIV-1 Subtype C Infection
Source: PLoS One. 2009 Nov 5;4(11):e7727. doi: 10.1371/journal.pone.0007727 (PMC2768328; doi:10.1371/journal.pone.0007727)
Supplement: Figure S8 — Alignment of translated amino acids in subject E-3430 at 8 time points from day 30 p/s to day 404 p/s. Sampling time of sequences is outlined in sequence name, and is shown in days p/s as a 3-digit number after the abbreviated patient code “E_”. For example, E_030_01 delineate sampling at day 30 p/s, sequence number 1, E_059_01 outlines sampling at day 59 p/s, sequence number 1, etc. Sequences originating from viral RNA template are delineated with “RNA” at the end of sequence name, while all other sequences were generated from proviral DNA template. Shown sequences are compared to the first sequence in alignment. Note that numbering above alignment represents sequences in subject E-3430, and does not correspond to Gag amino acid numbering of HXB2. (0.12 MB EPS) [file pone.0007727.s008.pdf]

Fig. S8. Subject E-3430. Alignment of translated amino acids.

```
                      10        20        30        40        50        60        70        80        90       100
             ....|....|....|....|....|....|....|....|....|....|....|....|....|....|....|....|....|....|....|....|
E_030_01     KIRLRPGGSKRYMLKHLVWASRELERFAINPGLLETGEGCKQIMKQLQPALQTGSEELRSLYNTVATLYCVHAEIEVQDTKEALDKIEEEQNKCQQKTQQ
E_030_02     ....................................................................................................
E_030_03     ....................................................................................................
E_030_04     ....................................................................................................
E_030_05     ....................................................................................................
E_030_06     ..........................................................................V.........................
E_030_07     ....................................................................................................
E_030_08     ....................................................................................................
E_030_09     ....................................................................................................
E_030_10     ....................................................................................................
E_030_11     ....................................................................................................
E_030_12     ....................................................................................................
E_030_13     ....................................................................................................
E_030_14     ....................................................................................................
E_030_15     ....................................................................................................
E_030_16     ....................................................................................................
E_030_17     ....................................................................................................
E_030_18     ....................................................................................................
E_030_19     ....................................................................................................
E_030_20     ........................G...........................................................................
E_030_21     ....................................................................................................
E_030_22     ....................................................................................................
E_030_23     ....................................................................................................
E_030_24     ....................................................................................................
E_059_01     ....................................................................................................
E_059_04     ....................................................................................................
E_059_05     ....................................................................................................
E_059_06     ............................................R.......................................................
E_059_15     ....................................................................................................
E_059_16     ....................................................................................................
E_059_17     ....................................................................................................
E_059_18     ....................................................................................................
E_059_19     ....................................................................................................
E_059_20     ....................................................................................................
E_150_01     ....................................................................................................
E_150_02     ..........K.........................................................................................
E_150_03     ..........K.........................................................................................
E_150_04     ....................................................................................................
E_150_05     ....................................................................................................
E_150_06     ..........K.........................................................................................
E_150_07     ..........K.........................................................................................
E_150_08     ....................................................................................................
E_150_10     ..........K.........................................................................................
E_243_01RNA  ..........K.........................................................................................
```

```
E_243_02RNA  ..........K.........................................................................................
E_243_03RNA  ..........K.........................................................................................
E_243_04RNA  ..........K..................................................................................G......
E_243_05RNA  ..........K..................................................................................G......
E_243_06RNA  ..K.......K.........................................................................................
E_274_01     ..........K.........................................................................................
E_274_02     ..........K..................................................................................G......
E_274_03     ..........K.........................................................................................
E_274_04     ..........K..................................................................................G......
E_274_05     ..........K.........................................................................................
E_274_06     ..........K..................................................................................G......
E_274_07     ..........K..................................................................................G......
E_274_08     ..........K..................................................................................S......
E_274_09     ..........K.........................................................................................
E_274_10     ..........K..................................................................................G......
E_305_01     ..........K..................................................................................G......
E_305_02     ..........K.........................................................................................
E_305_03     ..........K..................................................................................G......
E_305_04     ..........K..................................................................................G......
E_305_05     ..........K.........................................................................................
E_305_06     ..........K..................................................................................G......
E_305_07     ..........K..................................................................................G......
E_305_08     ..........K.........................................................................................
E_305_09     ..........K..................................................................................G......
E_305_10     ..........K..................................................................................G......
E_339_01     ..........K.........................................................................................
E_339_02     ..........K..................................................................................G......
E_339_03     ..........K..................................................................................G......
E_339_05     ..........K.........................................................................................
E_339_06     ..........K.........................................................................................
E_339_07     ..K.......K..................................................................................G......
E_339_09     ....................................................................................................
E_339_10     ..........K.........................................................................................
E_404_01     ..........K..................................................................................G......
E_404_02     ..........K..................................................................................G......
E_404_03     ..........K..................................................................................G......
E_404_04     ..........K..................................................................................G......
E_404_04RNA  ..........K.....................................................I...................................
E_404_05     ..........K.........................................................................................
E_404_05RNA  ..........K..................................................................................G......
E_404_06     ..........K..................................................................................G......
E_404_07     ..K.......K.........................................................................................
E_404_09     ..........K..................................................................................G......
E_404_09RNA  ..........K........................................R................................................
E_404_10     ..........K..................................................................................G......
E_404_10RNA  ..K.......K..................................................................................G......
```

```
E_404_12RNA   ..........K..............................................................E....R...............G......
E_404_13RNA   ..K.......K.........................................................................................
E_404_14RNA   ..........K..................................................................................G......
E_404_15RNA   ..........K..................................................................................G......
E_404_16RNA   ..........K..................................................................................G......
E_404_17RNA   ..........K..................................................................................G......
E_404_18RNA   ..........K..................................................................................G......
E_404_19RNA   ........N.K..................................................................R...............G......
E_404_20RNA   ..........K.........................................................................................

                     110       120       130       140       150       160       170       180       190       200
              ....|....|....|....|....|....|....|....|....|....|....|....|....|....|....|....|....|....|....|....|
E_030_01      AKEADGKVSQNYPIVQNLQGQMVHQAISPRTLNAWVKVIEEKGFNPEVIPMFTALSEGATPQDLNTMLNTVGGHQAAMQMLKDTINEEAAEWDRLHPVHA
E_030_02      ....................................................................................................
E_030_03      ....................................................................................................
E_030_04      ....................................................................................................
E_030_05      ....................................................................................................
E_030_06      ....................H.........P.....................................................................
E_030_07      ....................................................................................................
E_030_08      ....................................................................................................
E_030_09      ....................................................................................................
E_030_10      ....................................................................................................
E_030_11      ....................................................................................................
E_030_12      ....................................................................................................
E_030_13      ....................................................................................................
E_030_14      ....................................................................................................
E_030_15      ....................................................................................................
E_030_16      ....................................................................................................
E_030_17      ....................................................................................................
E_030_18      ....................................................................................................
E_030_19      ....................................................................................................
E_030_20      ....................................................................................................
E_030_21      ....................................................................................................
E_030_22      ....................................................................................................
E_030_23      ....................................................................................................
E_030_24      ....................................................................................................
E_059_01      ....................................................................................................
E_059_04      ....................................................................................................
E_059_05      ....................................................................................................
E_059_06      ....................................................................................................
E_059_15      ....................................................................................................
E_059_16      ....................................................................................................
E_059_17      ....................................................................................................
E_059_18      ....................................................................................................
E_059_19      ....................................................................................................
E_059_20      ....................................................................................................
```

```
E_150_01       ....................................................................................................
E_150_02       ....................................................................................................
E_150_03       ....................................................................................................
E_150_04       ....................................................................................................
E_150_05       ....................................................................................................
E_150_06       ....................................................................................................
E_150_07       ....................................................................................................
E_150_08       ....................................................................................................
E_150_10       ..........................L.........................................................................
E_243_01RNA    ..........................L.........................S...............................................
E_243_02RNA    ....................................................................................................
E_243_03RNA    ..........................L.........................................................................
E_243_04RNA    ..........................L.........................................................................
E_243_05RNA    ..........................L.........................................................................
E_243_06RNA    ..........................L.........................................................................
E_274_01       ..........................L.........................................................................
E_274_02       ..........................L.........................................................................
E_274_03       ..........................L.........................................................................
E_274_04       ..........................L.........................................................................
E_274_05       ..........................L.........................................................................
E_274_06       ..........................L.........................................................................
E_274_07       ..........................L.........................................................................
E_274_08       ..........................L.........................................................................
E_274_09       ..........................L.........................................................................
E_274_10       ..........................L.........................................................................
E_305_01       ..........................L.........................................................................
E_305_02       ..........................L.........................................................................
E_305_03       ..........................L.........................................................................
E_305_04       ..........................L.........................................................................
E_305_05       ..........................L.........................................................................
E_305_06       ..........................L.........................................................................
E_305_07       ..........................L.........................................................................
E_305_08       ..........................L.........................................................................
E_305_09       ..........................L.........................................................................
E_305_10       ..........................L.........................................................................
E_339_01       ..........................L.........................................................................
E_339_02       ..........................L.........................................................................
E_339_03       ..........................L.........................................................................
E_339_05       ..........................L.........................................................................
E_339_06       ..........................L.........................................................................
E_339_07       ..........................L.........................................................................
E_339_09       ..........................L.........................................................................
E_339_10       ..........................L.........................................................................
E_404_01       ..........................L.........................................................................
E_404_02       ..........................L.........................................................................
E_404_03       ..........................L.........................................................................
```

```
E_404_04       ..........................L..........................................................................
E_404_04RNA    ..........................L..........................................................................
E_404_05       ..........................L..........................................................................
E_404_05RNA    ..........................L..........................................................................
E_404_06       ..........................L..........................................................................
E_404_07       ..........................L..........................................................................
E_404_09       ..........................L..........................................................................
E_404_09RNA    ..........................L..........................................................................
E_404_10       ..........................L..........................................................................
E_404_10RNA    ..........................L..........................................................................
E_404_12RNA    ..........................L..........................................................................
E_404_13RNA    ..........................L..........................................................................
E_404_14RNA    ..........................L..........................................................................
E_404_15RNA    ..........................L..........................................................................
E_404_16RNA    ..........................L..........................................................................
E_404_17RNA    ..........................L..........................................................................
E_404_18RNA    ..........................L..........................................................................
E_404_19RNA    ..........................L..........................................................................
E_404_20RNA    ..........................L..........................................................................

                        210       220       230       240       250       260       270       280       290       300
               ....|....|....|....|....|....|....|....|....|....|....|....|....|....|....|....|....|....|....|....|
E_030_01       GPAAPGQIREPRGSDIAGTTSTLQEQIAWMTNNPAIPVGDIYKRWIILGLNKIVRMYSPVSILDIKQGPKEPFRDYVDRFFKTLRAEQATQDVKNWMTDT
E_030_02       ....................................................................................................
E_030_03       ....................................................................................................
E_030_04       ....................................................................................................
E_030_05       ....................................................................................................
E_030_06       .............................................................................A......................
E_030_07       ....................................................................................................
E_030_08       ....................................................................................................
E_030_09       ....................................................................................................
E_030_10       ....................................................................................................
E_030_11       ....................................................................................................
E_030_12       ....................................................................................................
E_030_13       ....................................................................................................
E_030_14       ....................................................................................................
E_030_15       ....................................................................................................
E_030_16       ....................................................................................................
E_030_17       ....................................................................................................
E_030_18       ....................................................................................................
E_030_19       ....................................................................................................
E_030_20       ....................................................................................................
E_030_21       ....................................................................................................
E_030_22       ....................................................................................................
E_030_23       ....................................................................................................
E_030_24       ....................................................................................................
```

```
E_059_01      ....................................................................................................
E_059_04      ....................................................................................................
E_059_05      ....................................................................................................
E_059_06      ....................................................................................................
E_059_15      ....................................................................................................
E_059_16      ....................................................................................................
E_059_17      ....................................................................................................
E_059_18      ....................................................................................................
E_059_19      ....................................................................................................
E_059_20      ....................................................................................................
E_150_01      ....................................................................................................
E_150_02      ....................................................................................................
E_150_03      ....................................................................................................
E_150_04      ....................................................................................................
E_150_05      ....................................................................................................
E_150_06      ....................................................................................................
E_150_07      ....................................................................................................
E_150_08      ....................................................................................................
E_150_10      ....................................................................-...............................
E_243_01RNA   .....A..............................................................................................
E_243_02RNA   ....................................................................................................
E_243_03RNA   ....................................................................................................
E_243_04RNA   ....................................................................................................
E_243_05RNA   ....................................................................................................
E_243_06RNA   .......L............................................................................................
E_274_01      ....................................................................................................
E_274_02      ....................................................................................................
E_274_03      ....................................................................................................
E_274_04      ....................................................................................................
E_274_05      ....................................................................................................
E_274_06      ....................................................................................................
E_274_07      ....................................................................................................
E_274_08      ....................................................................................................
E_274_09      ....................................................................................................
E_274_10      ....................................................................................................
E_305_01      ....................................................................................................
E_305_02      ....................................................................................................
E_305_03      ....................................................................................................
E_305_04      ....................................................................................................
E_305_05      ....................................................................................................
E_305_06      ............................................*.......................................................
E_305_07      ....................................................................................................
E_305_08      ....................................................................................................
E_305_09      ....................................................................................................
E_305_10      ....................................................................................................
E_339_01      ....................................................................................................
```

```
E_339_02     ....................................................................................................
E_339_03     ....................................................................................................
E_339_05     ....................................................................................................
E_339_06     ....................................................................................................
E_339_07     ....................................................................................................
E_339_09     ....................................................................................................
E_339_10     ....................................................................................................
E_404_01     ....................................................................................................
E_404_02     ....................................................................................................
E_404_03     ....................................................................................................
E_404_04     ....................................................................................................
E_404_04RNA  ....................................................................................................
E_404_05     ....................................................................................................
E_404_05RNA  ....................................................................................................
E_404_06     ....................................................................................................
E_404_07     ....................................................................................................
E_404_09     ....................................................................................................
E_404_09RNA  ....................................................................................................
E_404_10     ....................................................................................................
E_404_10RNA  ....................................................................................................
E_404_12RNA  ....................................................................................................
E_404_13RNA  ....................................................................................................
E_404_14RNA  ....................................................................................................
E_404_15RNA  ....................................................................................................
E_404_16RNA  ....................................................................................................
E_404_17RNA  ....................................................................................................
E_404_18RNA  ....................................................................................................
E_404_19RNA  ....................................................................................................
E_404_20RNA  ....................................................................................................

                       310       320       330       340       350       360       370       380       390       400
             ....|....|....|....|....|....|....|....|....|....|....|....|....|....|....|....|....|....|....|....|
E_030_01     LLVQNANPDCRNILRALGPGTTLEEMMTACQGVGGPSHKARVLAEAMSQTNQTNIMMQRNNFKGPRRIIKCFNCGKEGHLARNCRAPRKNGCWKCGKEGH
E_030_02     ....................A.....................................................................KC.........
E_030_03     ....................A.....................................................................K..........
E_030_04     ....................A.....................................................................KC.........
E_030_05     ....................A...............................................................................
E_030_06     ....................A...............................................................................
E_030_07     ....................A.....................................................................K..........
E_030_08     ....................A...............................................................................
E_030_09     ....................A...............................................................................
E_030_10     ....................A.....................................................................K..........
E_030_11     ....................A...............................................................................
E_030_12     ....................A...............................................................................
E_030_13     ....................A.....................................................................K..........
E_030_14     ....................A...............................................................................
```

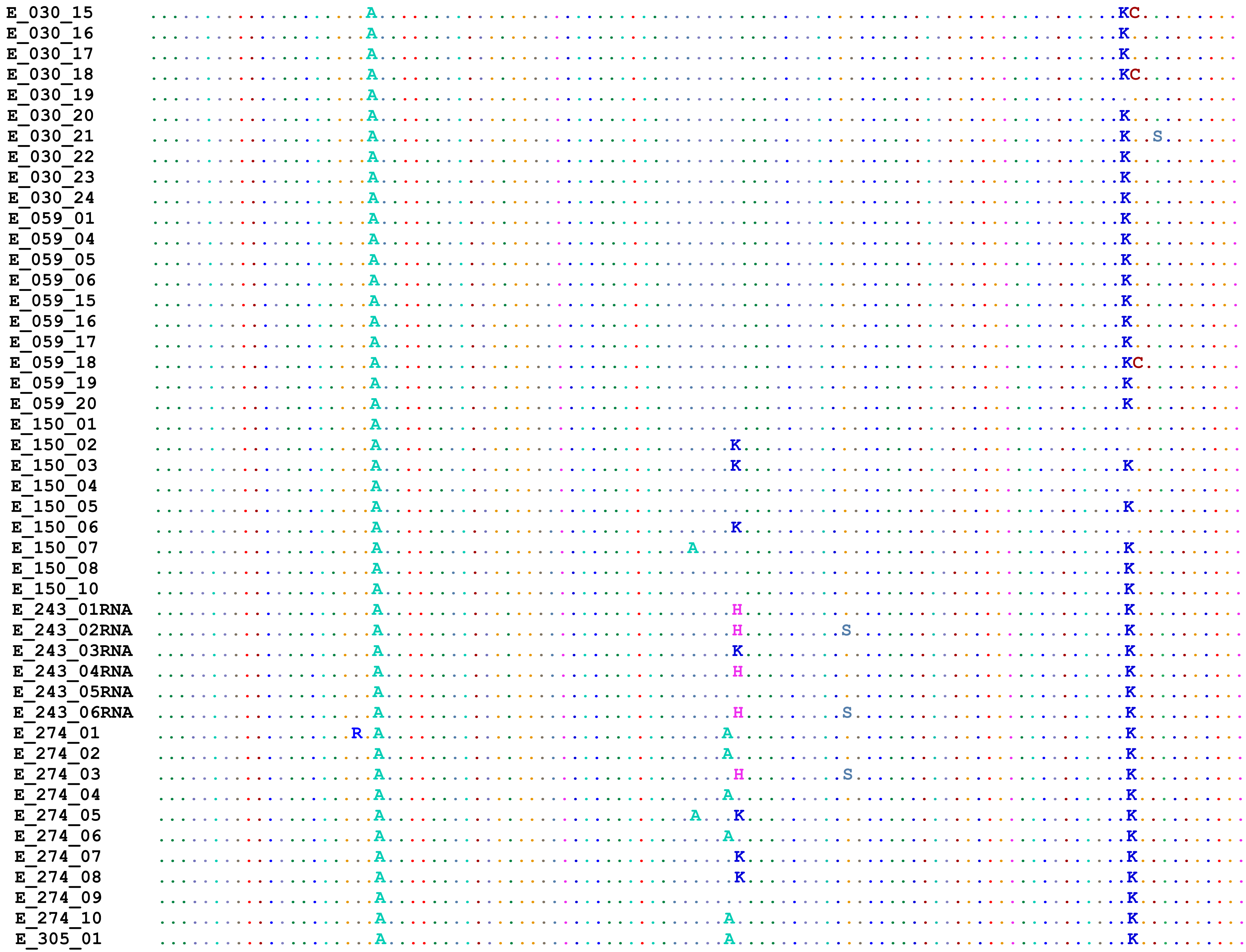
E_030_15 A KC
E_030_16 A K
E_030_17 A K
E_030_18 A KC
E_030_19 A
E_030_20 A K
E_030_21 A K S
E_030_22 A K
E_030_23 A K
E_030_24 A K
E_059_01 A K
E_059_04 A K
E_059_05 A K
E_059_06 A K
E_059_15 A K
E_059_16 A K
E_059_17 A K
E_059_18 A KC
E_059_19 A K
E_059_20 A K
E_150_01 A
E_150_02 A K
E_150_03 A K K
E_150_04 A
E_150_05 A K
E_150_06 A K
E_150_07 A A K
E_150_08 A K
E_150_10 A K
E_243_01RNA A H K
E_243_02RNA A H S K
E_243_03RNA A K K
E_243_04RNA A H K
E_243_05RNA A K
E_243_06RNA A H S K
E_274_01 R A A K
E_274_02 A A K
E_274_03 A H S K
E_274_04 A A K
E_274_05 A A K K
E_274_06 A A K
E_274_07 A K K
E_274_08 A K K
E_274_09 A K
E_274_10 A A K
E_305_01 A A K

```
E_305_02      ....................A...............................A....................................K..........
E_305_03      ....................A...............................A....................................K..........
E_305_04      ....................A...............................A....................................K..........
E_305_05      ....................A............................A.......................................K..........
E_305_06      ....................A...............................A....................................K..........
E_305_07      ....................A...............................A....................................K..........
E_305_08      ....................A...............................A....................................K..........
E_305_09      ....................A...............................A....................................K..........
E_305_10      ....................A................................K...................................K..........
E_339_01      ....................A................................K...................................K..........
E_339_02      ....................A...............................A....................................K..........
E_339_03      ....................A................................K...................................K..........
E_339_05      ....................A................................K...................................K..........
E_339_06      ....................A................................K...................................K..........
E_339_07      ....................A...............................A....................................K..........
E_339_09      ....................A....................................................................K..........
E_339_10      ....................A............................A...K...................................K..........
E_404_01      ....................A...............................A....................................K..........
E_404_02      ....................A...............................A....................................K..........
E_404_03      ....................A...............................A....................................K..........
E_404_04      ....................A...............................A....................................K..........
E_404_04RNA   ....................A...............................A....................................K..........
E_404_05      ....................A................................K...................................K..........
E_404_05RNA   ....................A...............................A....................................K..........
E_404_06      ....................A...............................A....................................K..........
E_404_07      ....................A................................K..............V....................K..........
E_404_09      ....................A...............................A....................................K..........
E_404_09RNA   ....................A...............................A....................................K..........
E_404_10      ....................A...............................A....................................K..........
E_404_10RNA   ....................A...............................A....................................K..........
E_404_12RNA   ....................A...............................A....................................K..........
E_404_13RNA   ....................A................................K..R................................K..........
E_404_14RNA   ....................A...............................A....................................K..........
E_404_15RNA   ....................A...............................A....................................K..........
E_404_16RNA   ....................A...............................A....................................K..........
E_404_17RNA   ....................A...............................A....................................K..........
E_404_18RNA   ....................A...............................A....................................K..........
E_404_19RNA   ....................A........Y.......................K...................................K..........
E_404_20RNA   ....................A................................K...................................K..........

                       410       420       430       440       450
              ....|....|....|....|....|....|....|....|....|....|...
E_030_01      QMKDCTERQANFLGKIWPSHKGRPGNFLQSRPEPSAPPAESFRFEETTPTPKQ
E_030_02      .....................................................
E_030_03      .....................................................
E_030_04      .....................................................
```

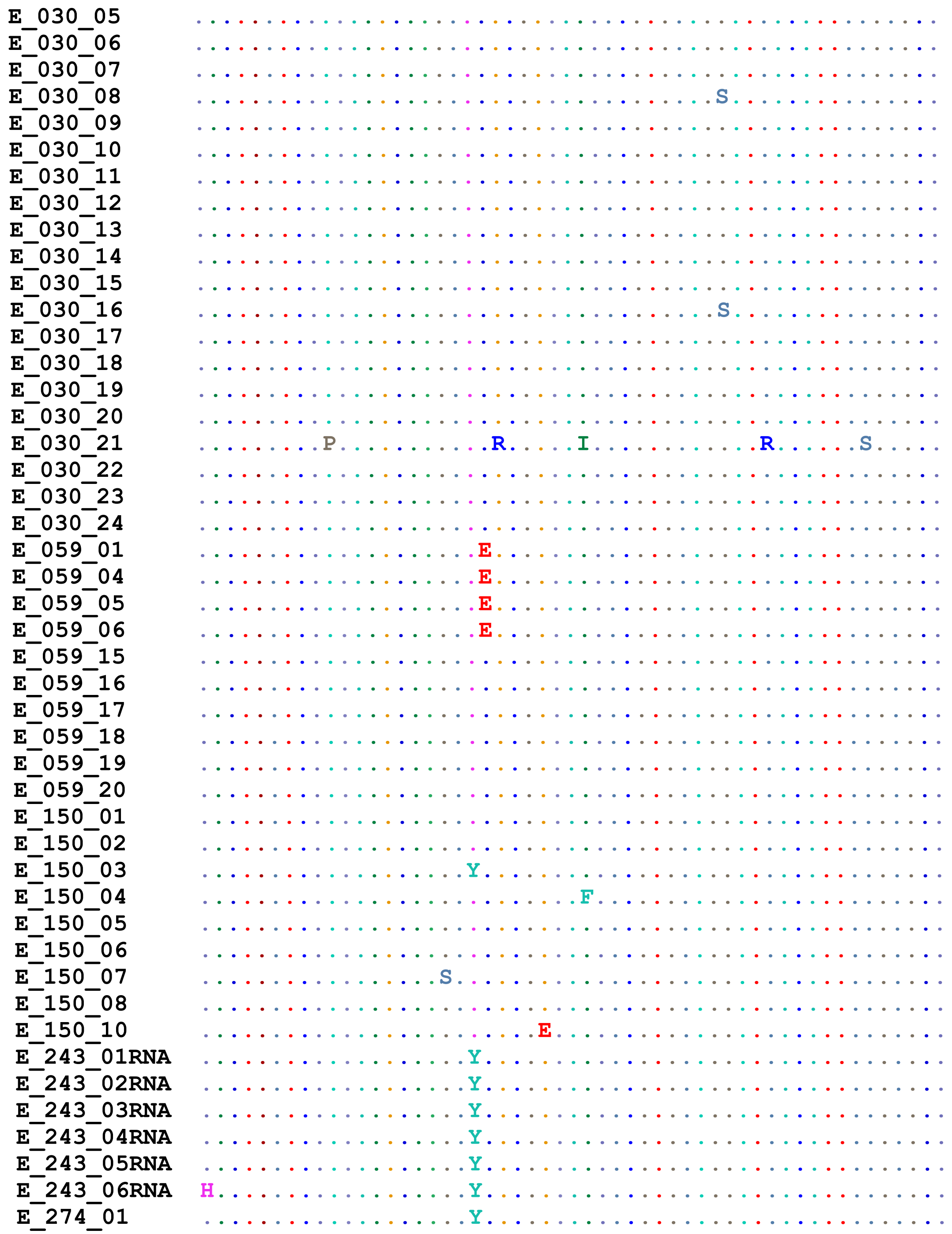
E_030_05
E_030_06
E_030_07
E_030_08 S
E_030_09
E_030_10
E_030_11
E_030_12
E_030_13
E_030_14
E_030_15
E_030_16 S
E_030_17
E_030_18
E_030_19
E_030_20
E_030_21 P R I R S
E_030_22
E_030_23
E_030_24
E_059_01 E
E_059_04 E
E_059_05 E
E_059_06 E
E_059_15
E_059_16
E_059_17
E_059_18
E_059_19
E_059_20
E_150_01
E_150_02
E_150_03 Y
E_150_04 F
E_150_05
E_150_06
E_150_07 S
E_150_08
E_150_10 E
E_243_01RNA Y
E_243_02RNA Y
E_243_03RNA Y
E_243_04RNA Y
E_243_05RNA Y
E_243_06RNA H Y
E_274_01 Y

```
E_274_02      ...................Y.................................
E_274_03      ...................Y.................................
E_274_04      ...................Y.................................
E_274_05      ...................Y.................................
E_274_06      ...................Y.................................
E_274_07      ...................Y.................................
E_274_08      ...................Y.................................
E_274_09      ...................Y.................................
E_274_10      ...................Y.................................
E_305_01      ...................Y.................................
E_305_02      ...................Y.................................
E_305_03      ...................Y.................................
E_305_04      ...................Y.................................
E_305_05      .................S...................................
E_305_06      ...................Y.................................
E_305_07      ...................Y.................................
E_305_08      ...................Y.................................
E_305_09      ...................Y.................................
E_305_10      ...................Y.................................
E_339_01      ...................Y.................................
E_339_02      ...................Y.................................
E_339_03      ...................Y.................................
E_339_05      ...................Y.................................
E_339_06      ...................Y.................................
E_339_07      ...................Y.................................
E_339_09      .....................................................
E_339_10      ...................Y.................................
E_404_01      ...................Y.................................
E_404_02      ...................Y.................................
E_404_03      ...................Y.................................
E_404_04      ...................Y.................................
E_404_04RNA   ...................Y.................................
E_404_05      ...................Y.................................
E_404_05RNA   ...................Y.................................
E_404_06      ...................Y.................................
E_404_07      ..................................................L..
E_404_09      ...................Y.................................
E_404_09RNA   ...................Y.................................
E_404_10      ...................Y.................................
E_404_10RNA   ...................Y.................................
E_404_12RNA   ...................Y.................................
E_404_13RNA   ...................Y.................................
E_404_14RNA   ...................Y.................................
E_404_15RNA   ...................Y.................................
E_404_16RNA   ...................Y.................................
E_404_17RNA   ...................Y.................................
```

```
E_404_18RNA   ...................Y.................................
E_404_19RNA   ...................Y.............................H...
E_404_20RNA   ...................Y.................................
```
